# Demographic Factors Influencing the Impact of Coronavirus-Related Misinformation on WhatsApp: Cross-sectional Questionnaire Study

**DOI:** 10.2196/19858

**Published:** 2021-01-30

**Authors:** Jay Amol Bapaye, Harsh Amol Bapaye

**Affiliations:** 1 Department Of Internal Medicine Rochester General Hospital Rochester, NY United States; 2 Foundation for Research and Education in Endoscopy Pune India; 3 Byramjee Jeejeebhoy Medical College and Sassoon General Hospital Pune India

**Keywords:** coronavirus, COVID-19, SARS–CoV–2, WhatsApp, social media, misinformation, infodemiology, infodemic, pandemic, medical informatics

## Abstract

**Background:**

The risks of misinformation on social networking sites is a global issue, especially in light of the COVID-19 infodemic. WhatsApp is being used as an important source of COVID-19–related information during the current pandemic. Unlike Facebook and Twitter, limited studies have investigated the role of WhatsApp as a source of communication, information, or misinformation during crisis situations.

**Objective:**

Our study aimed to evaluate the vulnerability of demographic cohorts in a developing country toward COVID-19–related misinformation shared via WhatsApp. We also aimed to identify characteristics of WhatsApp messages associated with increased credibility of misinformation.

**Methods:**

We conducted a web-based questionnaire survey and designed a scoring system based on theories supported by the existing literature. Vulnerability (K) was measured as a ratio of the respondent’s score to the maximum score. Respondents were stratified according to age and occupation, and K_mean_ was calculated and compared among each subgroup using single-factor analysis of variance and Hochberg GT2 tests. The questionnaire evaluated the respondents’ opinion of the veracity of coronavirus-related WhatsApp messages. The responses to the false-proven messages were compared using z test between the 2 groups: coronavirus-related WhatsApp messages with an attached link and/or source and those without.

**Results:**

We analyzed 1137 responses from WhatsApp users in India. Users aged over 65 years had the highest vulnerability (K_mean_=0.38, 95% CI 0.341-0.419) to misinformation. Respondents in the age group 19-25 years had significantly lower vulnerability (K_mean_=0.31, 95% CI 0.301-0.319) than those aged over 25 years (*P*<.05). The vulnerability of users employed in elementary occupations was the highest (K_mean_=0.38, 95% CI 0.356-0.404), and it was significantly higher than that of professionals and students (*P*<.05). Interestingly, the vulnerability of healthcare workers was not significantly different from that of other occupation groups (*P*>.05). We found that false CRWMs with an attached link and/or source were marked true 6 times more often than false CRWMs without an attached link or source (*P*<.001).

**Conclusions:**

Our study demonstrates that in a developing country, WhatsApp users aged over 65 years and those involved in elementary occupations were found to be the most vulnerable to false information disseminated via WhatsApp. Health care workers, who are otherwise considered as experts with regard to this global health care crisis, also shared this vulnerability to misinformation with other occupation groups. Our findings also indicated that the presence of an attached link and/or source falsely validating an incorrect message adds significant false credibility, making it appear true. These results indicate an emergent need to address and rectify the current usage patterns of WhatsApp users. This study also provides metrics that can be used by health care organizations and government authorities of developing countries to formulate guidelines to contain the spread of WhatsApp-related misinformation.

## Introduction

COVID-19, commonly referred to as the novel coronavirus disease, was first reported in Wuhan city, Hubei Province, in the People’s Republic of China in late December 2019 [1]. Since then, it has spread to over 3.4 million people across 212 countries around the world and claimed the lives of over 239,892 people as of May 2, 2020 [2]. The first case of COVID-19 in India was reported in the state of Kerala on January 30, 2020 [3], and as of May 2, 2020, the number of active COVID-19 cases was 26,167 with 1218 deaths reported [4]. A simple Google search about “coronavirus” yields over 5 billion results. These numbers are in line with the fact that COVID-19 has been declared as not only a pandemic but also an infodemic [5].

The established treatment options available for COVID-19 are limited, and the disease outcomes are worrisome. Such sensitive scenarios may lead people to believe or, even worse, act on information such as unverified remedies for COVID-19 without confirming its authenticity [6]. This might lead to serious adverse events, which mandates limiting misinformation on public platforms.

Social networking sites (SNS) are an important source of communication as well as information due to their easy accessibility, rapid transmission, simple user interface, dependability, and widespread outreach. Along with its advantages, social media also has a dark side of propagation of rumors or misleading information, to the extent that Goolsby [7] demonstrated that social media can be potentially used to orchestrate crime. Kumar et al [8] has defined misinformation as the spread of false information without the intent to deceive. Several studies have evaluated the resourcefulness of Facebook and Twitter during natural disasters and health care crises, including the COVID-19 pandemic [9-11]. Studies have also described problems related to misinformation on Facebook and Twitter, especially during health crises. Sharma et al [12] reported that misleading posts were far more popular than the posts dispersing accurate relevant public health information about the Zika virus disease. Kouzy et al [10] reported alarming rates of COVID-19–related misinformation being disseminated on Twitter [10]. The social media platform WhatsApp has over 2 billion users across 180 countries, enabling global peer-to-peer and mass communication [13], with over 400 million users in India alone [14]. One in two Indians received some sort of misinformation via WhatsApp or Facebook within the span of merely 30 days in the year 2019 [15]. This kind of misinformation, especially related to health care, on a platform with minimal surveillance such as WhatsApp could have far-reaching adverse consequences. Thus, efforts to better identify and potentially rectify this issue are crucial.

Dodda et al [16] reported that certain age groups could be more vulnerable to misinformation via WhatsApp. Our study aimed to identify specific demographic groups that may be more vulnerable towards misinformation via WhatsApp. Vulnerability of users towards content on WhatsApp may be influenced by several factors, ranging from the mere appearance of a message to different types of evidence included in a message [8].

Our study aimed to analyze the effect of evidence included in a message on the credibility of a message shared on WhatsApp.

## Methods

### Study Design

This study involved a web-based questionnaire survey conducted at Pune, Maharashtra, India. The study was specifically designed to perform a comparative analysis of vulnerability of user groups to misinformation based on a function of 9 parameters. Our methodology was novel and not based on the existing relevant literature.

### Research Model (Objective 1)

The study was designed to calculate a value K, defined as the risk of a WhatsApp user to get exposed to, accept, act on, or share unverified data regarding COVID-19 via WhatsApp. K was a value defined specifically for the purpose of this study. In a diverse population, several parameters may affect K. We identified 9 such parameters (P) framed by theories based on the existing literature, as detailed below.

#### P_1_—Duration of WhatsApp Usage Affects K

According to an article published in Business Today, India reported an 87% increase in the use of SNS during the first week of the COVID-19 lockdown as compared to the previous week [17]. Ünal [18] and Junco [19] reported a correlation between the duration of the use of SNS and the user’s investment of physical and psychological energies. In accordance with these reports, we posit that the duration of WhatsApp usage affects K.

#### P_2_—Priority of WhatsApp for Communication Affects K

Vosoughi et al [20] reported that even false information reaches more people if it is shared on a peer-to-peer basis rather than being broadcasted by a few users.

The social normative theory states that accepting and sharing content aligned with one’s peers’ beliefs is attractive regardless of its veracity [8,21], implying that users tend to believe more in information shared through peers. In the context of WhatsApp, sharing of information on a peer-to-peer basis is equivalent to its use as a social messenger. Hence, we posit that the priority of WhatsApp for communication affects K.

#### P_3_—Priority of WhatsApp for News and Information Affects K

Kumar and Shah [8] reported that the role of SNS for news and information has been on the rise as compared to traditional news sources. Out of 67% US adults using SNS as a source of news, 20% reported a relatively high usage frequency [22]. This confirms the dominance and dependence of SNS as news sources. Although SNS are great tools for disseminating information, they have a potential disadvantage of limited verification [23]. Thus, the level of priority placed in the use of SNS for news and information affects K. Hence, we posit that the priority of WhatsApp for news and information affects K and that the use of SNS for COVID-19–related updates affects K.

#### P_4_—Use of SNS for News Updates Regarding COVID-19 Affects K

Misinformation about COVID-19 is prevalent across all SNS [5]. Individuals using WhatsApp for COVID-19–related news updates are at risk of being exposed to misinformation and of propagating it via WhatsApp, thus affecting K. Even if a user does not use WhatsApp for news updates regarding COVID-19 but uses other SNS for the same purpose, they could still be exposed to COVID-19–related misinformation via these platforms. This misinformation could be further shared by the user via WhatsApp, thus affecting K.

#### P_5_—Trust Placed in COVID-19–Related Information Received via WhatsApp Affects K

The American Psychological Association defines trust as “reliance on or confidence in the dependability of someone or something” [24]. Reliance on or confidence in WhatsApp directly affects the acceptance of information provided therein. Hence, we posit that trust placed in COVID-19–related information received via WhatsApp affects K.

#### P_6_—Fact Check Rate Affects K

Misinformation has become a major menace on all SNS, including WhatsApp. SNS are reported to be untrustworthy during critical situations [25]. Multiple organizations such as boomlive.in [26] and factcheck.org [27] aid users to distinguish between true and false information received on SNS. Fact-checking could thus limit blind acceptance of false information and prevent its inadvertent dissemination. Actions based on such misinformation could also be prevented. Hence, we posit that the fact check rate affects K.

#### P_7_—Forward Rate Affects K

SNS like Facebook and Twitter implement determinants such as likes, comments, or shares of posts to analyze user engagement [19]. WhatsApp is primarily a messenger with a user interface designed specifically for communication; hence, determinants of user engagement of other SNS cannot be directly applied to it. The user interface of WhatsApp allows people to be members of groups comprising as many as 256 persons. Users can thus forward messages in a peer-to-peer fashion and simultaneously to multiple groups. This allows WhatsApp users the potential to rapidly disseminate information, regardless of its authenticity, to a staggering number of individuals. Hence, we posit that the forward rate of WhatsApp messages affects K.

#### P_8_—User’s Ability to Discern a WhatsApp Message as True or False Affects K

Several studies have conducted experiments to measure the ability of humans to detect false information and have shown that humans are not particularly good at discerning false information from true. Kumar and Shah [8] reported that humans correctly identified a hoax merely 66% of times. False information would not have any influence if readers were able to tell that it is false [8].

Hence, we posit that the user’s ability to discern a WhatsApp message as true or false affects K.

#### P_9_—Actions Taken in Response to a Message Affects K

SNS have the potential to motivate users to act based on any information provided to them. For instance, a rumor on WhatsApp claiming that public transport will be made available to transport over 1000 workers back to their hometowns resulted in a gathering of 700-800 people on a railway station in India [28]. Based on these reports, we posit that actions taken in response to a message affects K.

These 9 parameters P_1_-P_9_ were used to formulate the study questionnaire.

### Data Collection

We conducted a web-based questionnaire survey. Its design was influenced by models used by Oyero et al [23] and Dodda et al [16]. The questionnaire was created using Google Forms in 2 languages—English and vernacular (Marathi), as the target population was fluent in either of these languages (see Multimedia Appendix 1).

It consisted of 28 questions: 5 about demographics, 12 regarding general and COVID-19–related WhatsApp usage, and 10 regarding coronavirus related WhatsApp messages (CRWMs) that the respondents were asked to identify as true or false. These 10 messages were selected randomly from a pool of 30 WhatsApp messages commonly shared in India, sourced from AlJazeera, Reuters, and Google Images [29,30]. These questions were specifically included to reduce the risk of biased reporting through the survey and increase its accuracy. The final question was open-ended and enquired the respondent’s opinion about WhatsApp as an information tool in the current COVID-19 pandemic. Of the 28 questions, 21 (Q_K_) were framed to evaluate the 9 parameters (P_1_-P_9_) that affect K as detailed in the section “Research Model (Objective 1).” The questionnaire was initially distributed among a WhatsApp group comprising users from diverse demographics. The first 10 respondents were consulted to check for errors and obtain feedback regarding understandability of the selected questions. Necessary modifications were made accordingly, and pilot responses were excluded from the analysis. The final questionnaire was shared on the following social media platforms: WhatsApp, Facebook, Twitter, Instagram, and LinkedIn. All responses were voluntary and anonymous. The survey was conducted from April 8 to April 13, 2020. Data were compiled using Microsoft Excel (Microsoft Corp.).

### Scoring System for Calculation of K

The 21 questions (Q_K_) evaluating the 9 parameters (P_1_-P_9_) were sorted into 9 groups based on the parameter being tested (Table 1). Numerical scores were assigned to the response options of each of the 9 question groups. Responses that were assigned a score “0” had no impact on K. For other responses, higher the value of the score, greater was the impact of the response on K.

**Table 1:** Scoring system for calculation of K values.

Question group no.^a^	Response options (score)				
1. Duration of WhatsApp use	0-30 minutes (1)	30 minutes to 1 hour (2)	1-2 hours (3)	>2 hours (4)	N/A^b^
2. Communication^c^	Low (1)	Moderate (2)	High (3)	N/A	N/A
3. Information^d^	Low (1)	Moderate (2)	High (3)	N/A	N/A
4. Source for COVID-19–related updates^e^	Neither social media nor WhatsApp (0)	Social Media but no WhatsApp (2)	WhatsApp (4)	N/A	N/A
5. Trust^f^	0% (0)	25% (1)	50% (2)	75% (3)	100% (4)
6. Forwards^g^	0-2 (1)	3-5 (2)	6-8 (3)	More than 8 (4)	N/A
7. Fact-check^h^	75-100% (1)	50-75% (2)	25-50% (3)	0-25% (4)	Never (5)
8. Incorrect responses^i^	0-2 (1)	3-5 (2)	6-8 (3)	More than 8 (4)	N/A
9. Actions or opinions^j^	Never considered using (0)	Considered but not used (1)	Used once (2)	Using regularly and recommending (3)	N/A

^a^Question group numbers 1-9 represent questions testing parameters P_1_-P_9_, respectively.

^b^Not applicable.

^c^Priority of WhatsApp usage for communication.

^d^Priority of WhatsApp usage for information.

^e^Sources used by respondents to stay updated with COVID-19–related information.

^f^Trust placed in COVID-19–related WhatsApp messages.

^g^Number of forwards about COVID-19 per day.

^h^Percentage of COVID-19–related messages that were fact-checked.

^i^Number of incorrect responses provided while discerning a WhatsApp message as true or false.

^j^Actions taken by respondents in line with unverified information received via WhatsApp.

The 7 parameters (P_1_-P_7_) were tested by question groups 1-7, each consisting of 1 question. Each of these questions was assigned an individual score. Question group 4 was assigned a different scoring system as it recorded the source for COVID-19–specific information, which has a stronger impact on K. COVID-19–related misinformation available on traditional news sources is negligible. Hence, respondents referring to only these sources (ie, sources other than social media and WhatsApp for COVID-19–related information) are not at risk of misinformation. As this does not affect K, the score assigned to this response was “0.” Respondents referring to WhatsApp as a source of COVID-19–related information have a high impact on K; their responses were therefore assigned a score of “4.” Those referring to social media but not WhatsApp still carry the risk of sharing misinformation via WhatsApp, thereby moderately impacting K; a score of “2” was therefore assigned for their responses.

A total of 10 questions out of the 21 Q_K_ tested user ability to discern a WhatsApp message as true or false (P_8_). They were grouped together in question group 8. The total number of incorrect responses was calculated for these 10 questions, and respondents were thus assigned scores.

Four of the 21 Q_K_ questions recorded the actions taken by a user in response to unverified treatment options (herbal, homeopathic, ayurvedic, and home remedies) for COVID-19 received via WhatsApp. These questions were grouped together as they measured P_9_. Taking an action in response to misinformation can lead to adverse health effects. A higher risk is therefore anticipated in this case in comparison with only exposure, acceptance, or sharing of misinformation. Since each of the 4 questions had a higher impact on K, they were scored individually.

One numerical score represented each question group (1-8), and 4 scores represented question group 9. A total of 12 scores were thus recorded, and their sum was calculated (S_cal_) for each respondent. The maximum possible score for any respondent was 46 (S_max_). The scoring system intended to compare subgroups of the same sample. S_cal_ of one respondent in relation to the S_cal_ of another respondent was measured, whereas the standalone value of S_cal_ had negligible relevance.

### Classification of Age and Occupation Subgroups

The study sample was classified into 6 groups according to the respondents’ age in years: under 18, 19-25, 26-35, 36-50, 51-65, and over 65. In addition, occupations of working adults were classified based on The International Standard Classification of Occupations [31]. Students and retired individuals were considered as separate groups.

This study was focused on WhatsApp usage during the COVID-19 pandemic—a global health care crisis. Therefore, health care workers (HCWs) were considered a separate occupation subgroup, as their usage patterns could have been unique.

### Computation of K

K was calculated as a ratio S_cal_/S_max_ for each respondent, and K_mean_ (±95% CI) was calculated for each age and occupation subgroup. K_mean_ was compared for age groups and occupations separately. Since K_mean_ was compared within the same sample, relative values of K_mean_ and not its absolute value were relevant in this study.

### Research Model (Objective 2)

Dodda et al [32] demonstrated that the presence of background evidence may increase the credibility of a message on WhatsApp. The Merriam-Webster dictionary defines credibility as “the quality or power of inspiring belief” [32]. In this study, we aimed to analyze factors that could affect the credibility of messages on WhatsApp during the COVID-19 pandemic.

The questionnaire included 10 commonly shared WhatsApp messages related to COVID-19, which the respondents were asked to rate as follows: definitely true, maybe true, maybe false, or definitely false. These were the same 10 questions that were used to test P8. The authenticity of these 10 messages was confirmed with data from authorized sources [29,30,33-44]. Of the 10 messages analyzed, 8 were false messages; these were selected and subcategorized as those with background evidence containing link or source: (N_s_) and those without: (N_x_).

The total number of instances that a false message was marked by the respondents as definitely true or maybe true was measured in each group (N_S_ and N_X_). The number of such instances was recorded as a_s_ in group N_S_ and a_x_ in group N_X_.

a_s_/N_s_=Number of instances that a false message was marked definitely true or maybe true for every message with background evidence.

a_x_/N_x_=Number of instances that a false message was marked definitely true or maybe true for every message without background evidence.

Comparison of these ratios detected whether an association exists between the presence of background evidence in a false WhatsApp message and the number of instances of it being marked as true.

### Statistical Analyses

All calculations were performed on SPSS software (Build no. 1.0.0.1347; IBM Corp.). The level of significance was set at *P*=.05 for all variables.

For research objective 1, a single-factor analysis of variance (ANOVA) was applied to compare K_mean_ values in the age and occupation subgroups separately [45]. Hochberg GT2 test was conducted to perform a post hoc analysis since variances were homogenous according to Hartley’s F_max_ test and sample sizes in each subgroup were different [46,47].

For research objective 2, a_s_/N_s_ and a_x_/N_x_ values were calculated and compared using 2-tailed z-test.

## Results

### Demographic Characteristics of the Study

We obtained a total of 1191 responses to our survey. Of these, 54 responses were not considered as the respondents either did not use WhatsApp or were not presently living in India. These responses were eliminated, and the remaining 1137 responses obtained from respondents across 20 Indian states and union territories were further analyzed. Demographic characteristics of the study sample are described in Table 2. No specific measures were taken to control the demographics of the respondents. Hence, the sample distribution across subgroups was varied.

**Table 2:** Demographic characteristics of the study sample.

Demographic		Value (N=1137), n (%)
**Age (years)**		
	Below 18	25 (2.20)
	19-25	395 (34.74)
	26-35	160 (14.07)
	36-50	291 (25.59)
	51-65	235 (20.67)
	Above 66	31 (2.73)
**Gender**		
	Men	648 (56.99)
	Women	488 (42.92)
	Gender fluid	1 (0.09)
**Occupation**		
	HCW^a^	291 (25.59)
	Other professionals	276 (24.27)
	Manager	18 (1.58)
	Service and sales	89 (7.83)
	Elementary occupation	72 (6.33)
	Clerical occupation	7 (0.62)
	Student	345 (30.34)
	Retired and unemployed	39 (3.43)

^a^ HCW: health care worker.

Of the 1137 respondents, 648 (56.99%) were men, 488 (42.92%) were women, and 1 (0.09%) was gender fluid. Age group distribution showed that 25 of the 1137 (2.20%) respondents were under 18 years, 395 (34.74%) were between 19 and 25 years, 160 (14.07%) were between 26 and 35 years, 291 (25.59%) were between 36 and 50 years, 235 (20.67%) were between 51 and 65 years, and 31 (2.73%) were over 65 years. Occupation-wise distribution showed 345 of the 1137 (30.34%) were students, 291 (25.59%) were HCWs, 276 (24.27%) were professionals, 89 (7.83%) were service and sales workers, 72 (6.33%) were involved in elementary occupations, 39 (3.43%) were retired and unemployed, 18 (1.58%) were managers, and 7 (0.62%) were clerical workers.

### Results for Objective 1

Usage patterns of WhatsApp in the sample population are detailed in Table 3. Active use of WhatsApp as a source of information regarding COVID-19 was confirmed by 355 of the 1137 (31.22%) respondents. Moreover, 657 (57.78%) respondents demonstrated trust in CRWMs, of which 139 (21.15%) trusted more than 50% of CRWMs. The presence of an attached link and/or source demonstrated an increase in CRWM credibility for 859 of the 1137 (75.55%) respondents. Furthermore, of the 1137 respondents, 151 (13.28%) never fact-checked CRWMs before forwarding them and 164 (14.43%) forwarded 3 or more CRWMs per day. The various actions taken or considered in response to CRWMs are detailed in Table 4.

**Table 3:** WhatsApp usage patterns in the context of COVID-19.

Variable		Value (N=1137), n (%)
**Duration of WhatsApp use**		
	0-30 minutes	136 (11.96)
	30 minutes to 1 hour	327 (28.76)
	1-2 hours	391 (34.39)
	>2 hours	283 (24.89)
**WhatsApp as a source of information regarding COVID-19 **		
	Yes	355 (31.22)
	No	782 (68.78)
**Trust in CRWM^a^ (%)**		
	0	479 (42.13)
	25	519 (45.65)
	50	121 (10.64)
	75	16 (1.41)
	100	2 (0.18)
**Factors accompanying CRWM^b^ **		
	Attached link and/or source	859 (75.55)
	Sender’s authenticity	374 (32.89)
	Attached multimedia	77 (6.77)
	None of the above	164 (14.42)
**Fact-check rate^c^ (%) **		
	Never	151 (13.28)
	1-25	114 (10.03)
	25-50	73 (6.42)
	50-75	132 (11.61)
	75-100	667 (58.66)
**Forward rate^d^**		
	0-2	973 (85.58)
	3-5	83 (7.30)
	6-8	26 (2.29)
	More than 8	55 (4.84)

^a^CRWM: COVID-19–related WhatsApp message.

^b^Users could select more than 1 factor which increased their trust in a CRWM.

^c^CRWMs fact-checked before forwarding.

^d^CRWMs forwarded per day.

**Table 4:** Actions taken in response to information regarding COVID-19 received vis WhatsApp.

Actionable measures		Value (N=1137), n (%)
**Social distancing **		
	Never considered using	46 (4.05)
	Considered but not used	138 (12.14)
	Used once	291 (25.59)
	Using regularly and recommending	662 (58.22)
**Masks **		
	Never considered using	50 (4.40)
	Considered but not used	188 (16.53)
	Used once	329 (28.94)
	Using regularly and recommending	570 (50.13)
**Allopathic remedies **		
	Never considered using	449 (39.49)
	Considered but not used	398 (35)
	Used once	131 (11.52)
	Using regularly and recommending	159 (13.98)
**Herbal remedies **		
	Never considered using	776 (68.25)
	Considered but not used	271 (23.83)
	Used once	58 (5.10)
	Using regularly and recommending	32 (2.81)
**Ayurvedic remedies **		
	Never considered using	740 (65.08)
	Considered but not used	302 (26.56)
	Used once	62 (5.45)
	Using regularly and recommending	33 (2.9)
**Homeopathic remedies**		
	Never considered using	788 (69.31)
	Considered but not used	268 (23.57)
	Used once	60 (5.28)
	Using regularly and recommending	21 (1.85)
**Home remedies**		
	Never considered using	803 (70.62)
	Considered but not used	200 (17.59)
	Used once	88 (7.74)
	Using regularly and recommending	46 (4.05)

Recommended practices such as social distancing and use of masks were not being followed by 184 (16.19%) and 238 (20.93%) of the 1137 respondents, respectively. Responses regarding the use of unverified treatment options revealed that 90 of the 1137 (7.91%) respondents had used herbal, 81 (7.13%) had used homeopathic, 95 (8.35%) had used ayurvedic, and 134 (11.79%) had used home remedies at least once.

**Figure 1:**
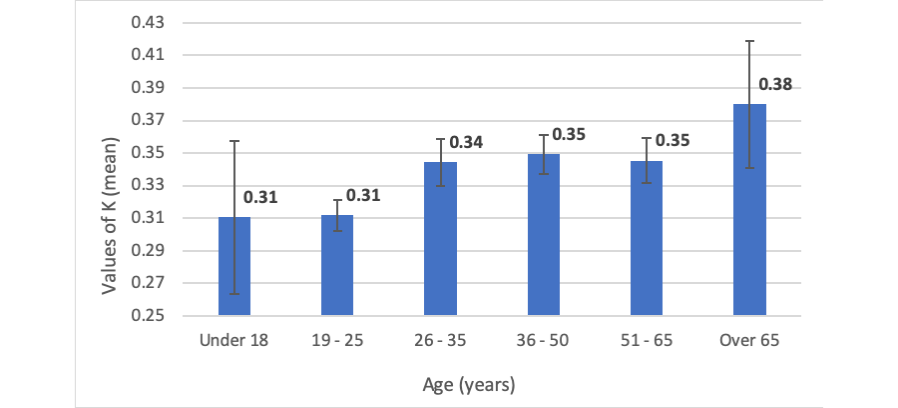
Graphical representation of K_mean_ values (± 95% CI) across age subgroups.

K_mean_ value was found to be the lowest for the under-18 years age subgroup (0.31, 95% CI 0.264-0.356) and the highest for the over-65 years age subgroup (0.38, 95% CI 0.341-0.419) (Figure 1). Statistically significant differences were found among K_mean_ values for the 6 age subgroups (*P*<.001, single-factor ANOVA). Post hoc analysis revealed that respondents in the age group 19-25 years had a significantly lower K_mean_ value (0.31, 95% CI 0.301-0.319) than all respondents aged over 25 years (Table 5).

**Table 5:** Post hoc comparisons (*P* values) among Kmean values of age subgroups using Hochberg GT2 test.

Age group (years)	Under 18	19-25	26-35	36-50	51-65	Over 65
Under 18	>.99	>.99	.85	.65	.80	.15
19-25	—^a^	>.99	.009	0	.001	.005
26-35	—	—	>.99	>.99	>.99	.68
36-50	—	—	—	>.99	>.99	.82
51-65	—	—	—	—	>.99	.68
Over 65	—	—	—	—	—	>.99

^a^Not applicable.

**Figure 2:**
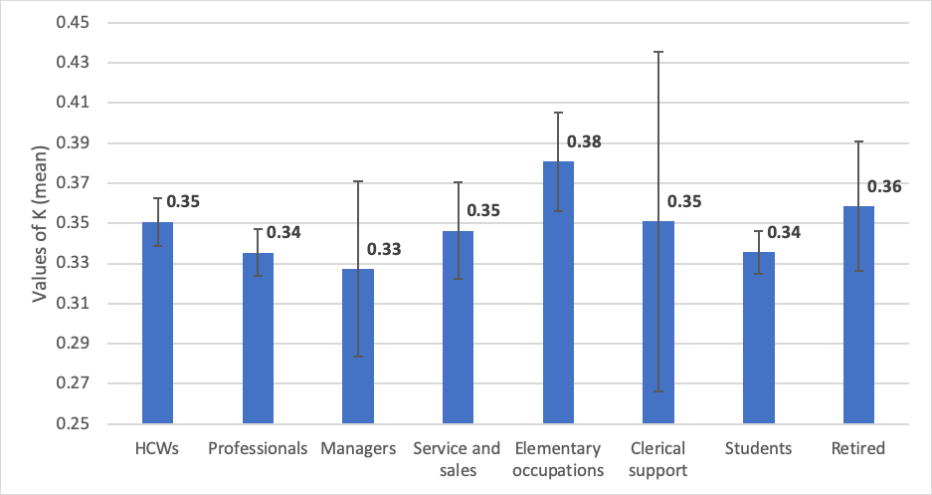
Graphical representation of K_mean_ values (± 95% CI) across occupation subgroups.

K_mean_ value was the lowest for managers (0.33, 95% CI 0.286-0.374) and the highest for elementary occupations (0.38, 95%, CI 0.356-0.404) (Figure 2). Statistically significant differences were observed among K_mean_ values measured for the 8 occupation groups (*P*=.023, single-factor ANOVA). Post hoc analysis revealed a significantly higher K_mean_ value in the elementary occupation subgroup than in the professionals and students’ subgroups (Table 6).

**Table 6:** Post hoc comparisons (*P* values) among Kmean values of occupation subgroups using Hochberg GT2 test.

Group	HCW^a^	Professionals	Service^b^	Managers	Elementary^c^	Clerical^d^	Students	Retired
HCW	>.99	.87	>.99	>.99	.52	>.99	.83	>.99
Professionals	—^e^	>.99	>.99	>.99	.02	>.99	>.99	.99
Service	—	—	>.99	>.99	.62	>.99	>.99	>.99
Managers	—	—	—	>.99	.74	>.99	>.99	>.99
Elementary	—	—	—	—	>.99	>.99	.02	>.99
Clerical	—	—	—	—	—	>.99	>.99	>.99
Students	—	—	—	—	—	—	>.99	.997
Retired	—	—	—	—	—	—	—	>.99

^a^HCW: health care worker.

^b^Service and sales workers.

^c^Elementary occupations.

^d^Clerical support workers.

^e^Not applicable.

### Results for Objective 2

**Figure 3:**
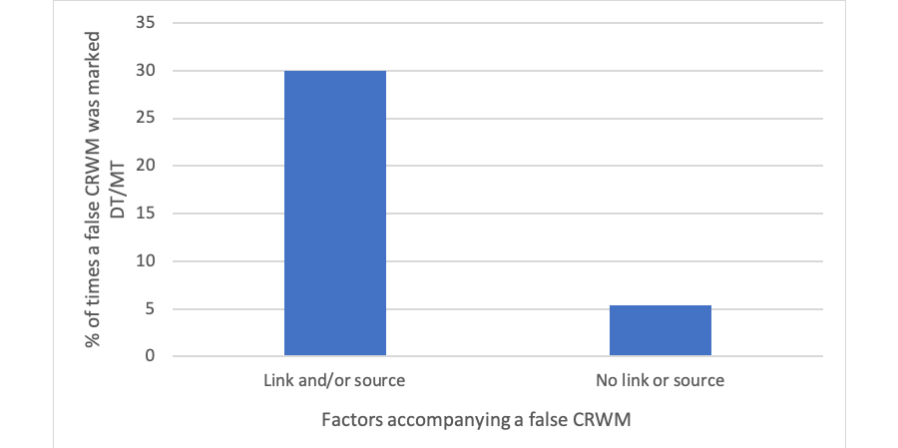
Number of times a false CRWM was marked as true for every false CRWM with and without background evidence (link and/or source). CRWM: coronavirus-related WhatsApp message, DT: definitely true, MT: maybe true.

Presence of background evidence in a false message demonstrated a significant impact on the perceived credibility of the WhatsApp user (a_s_/N_s_=0.299 vs a_x_/N_x_=0.053; *P*<.001, 2-tailed *z* test). The number of times false messages with background evidence were marked definitely true or maybe true was significantly (nearly 6 times) higher than that for false messages without background evidence (Figure 3).

## Discussion

COVID-19 has caused an unprecedented biopsychosocial crisis. It has been declared not only as a pandemic but also as a worldwide infodemic [5]. Mentions about coronavirus in the form of news, updates, and especially misinformation on SNS are rising exponentially. In this study, we broadly analyzed factors influencing misinformation related to the disease (COVID-19) on WhatsApp. van Velsen et al [25] studied the reliability of Facebook and Twitter during an infective health crisis. They concluded that the majority of their study sample did not find these platforms to be reliable sources of information, and only 11% of the participants used them passively for information [25]. However, 39.92% (454/1137) of our sample considered WhatsApp as a useful information tool during this health crisis. Moreover, our study demonstrates active WhatsApp use by only 31.22% (355/1137) of the sample for COVID-19–related information. This finding suggests an almost 4-fold increase in reliance of individuals on SNS, particularly WhatsApp for news and information in recent times. Although trust levels in traditional sources of news have been higher, 57.78% (657/1137) of our study sample demonstrated a certain level of trust in CRWMs. With every 1 in 2 Indians receiving some false news on WhatsApp or Facebook, the consequences of the trust placed in these platforms could be serious [15].

Community preventive measures against COVID-19, particularly social distancing and using face masks, have proven to be effective and have been strongly recommended by the Government of India [48]; however, our study demonstrated that 16.18% (184/1137) and 20.93% (238/1137) of our study participants, respectively, did not implement these measures. This finding indicates that a considerable proportion of individuals demonstrate reluctance towards authorized recommendations.

Currently, no pharmacological agent has been proven to reduce mortality in COVID-19 cases [6]. Nevertheless, our findings confirmed that 1.85%-7.74% of the respondents were using or had used some form of complementary or alternative medicines for COVID-19 (Table 4). This finding suggests an interesting paradox wherein there is reluctance to accept evidence-based recommendations in favor of easy acceptance of novel but unverified remedies. This could result in serious health-related adverse events [49].

In addition, our study identified certain age groups were at a higher risk of getting influenced by COVID-19–related misinformation via WhatsApp. In particular, users over 65 years demonstrated the highest vulnerability to misinformation, whereas those under 18 years demonstrated the lowest. Vulnerability to misinformation progressively increased with users’ age. Limited knowledge of fact-checking resources, unawareness about existing misinformation on WhatsApp, and positive reinforcement from echo chambers could possibly explain these results [8]. This is particularly worrisome considering that older users tend to be more susceptible to the adverse effects of unsupervised remedies and are at a higher risk of severe COVID-19. Dodda et al [16] reported that WhatsApp users aged below 20 years and those above 50 years were the most susceptible to fake news. In contrast, our study findings showed that users aged under 25 years were the least susceptible to misinformation. Users aged between 19 and 25 years were at a significantly lower risk than those over 25 years (*P*<.05). Users in this age group tend to be well versed with SNS, including WhatsApp. Awareness about fact-checking resources and existence of misinformation on WhatsApp could possibly have reduced their vulnerability.

Among the different occupation groups, those employed in elementary occupations demonstrated the highest vulnerability to false news (K_mean_=0.38). These users were significantly more vulnerable to misinformation than students and professionals (K_mean_=0.34 for both, *P*<.05). A possible explanation for this observation could be the lower levels of education among the elementary occupation subgroup. Managers demonstrated the least vulnerability among the sample. However, a statistically significant difference could not be established, presumably due to the small sample size of this subgroup.

The vulnerability among HCWs was found to be consistent with all other occupation groups. During a health care crisis like the COVID-19 pandemic, HCWs are considered reliable sources of information by the community. Kumar et al [8] concluded that if false information is purposefully made to appear genuine, it can deceive trained and casual readers alike. COVID-19 is an evolving global health crisis wherein the mortality rate is high, established treatment options are limited, and HCWs are at a high risk of contracting the disease [6,50]. This vulnerability, coupled with a desire to treat patients, could possibly lead even HCWs to accept novel but unverified information. Such medical misinformation inadvertently propagated by HCWs may have unbeknownst consequences on the community.

Dodda et al [16] reported that background evidence and trust in organizations and individuals can make people believe in information received on SNS. Kumar et al [8] reported that that lengthy and well-referenced hoaxes were frequently misjudged to be true. In our study, for more than half the sample (659/1137, 57.95%), an attached link or source was a factor associated with increasing trust in a CRWM. Presence of background evidence was strongly associated with an erroneously higher trust of a false CRWM (*P*<.001). This finding indicates that individuals are likely to place emphasis on the presence of background evidence in a false CRWM in addition to its content while judging its veracity. Identification of such factors and ways to limit seemingly credible misinformation on SNS are pertinent questions that need to be evaluated in this digital age.

SNS usage patterns in developing countries are broadly comparable, although regional preferences regarding the choice of SNS may be different [51]. WhatsApp is a popular SNS globally with over 2 billion users worldwide, of which 400 million users are from India [13,14]. In our web-based survey study on WhatsApp users from India, all survey responses obtained were anonymous and voluntary. The study sample was representative of all age groups and majority of the occupation groups. We, therefore, are of the opinion that the findings of this study are representative of WhatsApp usage patterns in developing countries.

Our study had certain limitations. First, it was a survey-based study wherein data was self-reported; therefore, the sample may be tainted by observer bias (Hawthorne effect [52]). Second, the number of respondents in certain age and occupation subgroups were fewer, which could increase the risk of type 2 error during subgroup analysis. Third, the respondents’ opinions regarding CRWMs may have been formed through sources other than WhatsApp. The questionnaire was specifically designed to include the terms “WhatsApp” and “coronavirus” in the questions to address this limitation. Fourth, this study tested 4 message-based factors that could affect users’ trust in CRWMs. However, it was possible that other personal, regional, or community-based factors could also affect the users’ trust. To ensure generalizability of the results of this study, we chose not to analyze these belief systems. Fifth, the research model has limited support from the existing literature owing to its specific nature. It could be implemented in further similar studies to improve its validity. Finally, although well-referenced, the parameters P_1_-P_9_ used in this study are based on theories supported by the existing literature; hence, the results should be interpreted as correlational.

The results of this study present several challenges regarding the current COVID-19 pandemic for WhatsApp, fact-checking bodies, health organizations, and government authorities in developing countries. Cuan-Baltazar et al [53] found that the use of the internet during a pandemic is a risk to public health and recommend that the authorities should develop strategies to regulate online health information without censuring the public. Disasters mandate the circulation of timely information, which may at times compromise its genuineness. SNS have traditionally faced the issue of credibility at the cost of rapid transmission of information [23,54]. This may reduce the resourcefulness of a highly efficient communication tool such as WhatsApp, as seen in recent trends of decreasing trust in SNS in developed countries [51,55].

To address these challenges, health and government authorities in developing countries could collaborate with WhatsApp to develop methods to authenticate and tamper-proof messages from official sources. Leadership in health care organizations could actively work towards addressing digital awareness among health care workers who are the anchor points of information for the rest of the community. Fact-checking organizations could increase their presence on and integrate with SNS to improve their resourcefulness. Authorities could undertake awareness campaigns to educate users of SNS to recognize misinformation. WhatsApp could allow users to report messages containing suspected misinformation to allow necessary measures to be undertaken. WhatsApp, if used as a platform to broadcast validated information to a userbase as large as 400 million while providing measures enabling them to discern genuine news from misinformation, could revolutionize disaster management in a developing country.

In conclusion, our study analyzed factors influencing COVID-19–related misinformation among WhatsApp users in a developing country. Older adults (above 65 years) were more vulnerable to misinformation circulated on WhatsApp, as were individuals employed in elementary occupations. HCWs were also not spared from the influence of misinformation and were found to be as vulnerable as any other occupations. Presence of background evidence in a false CRWM was strongly linked to an increase in its credibility. These findings may provide important insights to health organizations and government authorities of developing countries to formulate suitable guidelines to contain the COVID-19 infodemic. Further experimental studies about parameters tested in this study could be considered.

## Appendix

Multimedia Appendix 1 Study questionnaire.

## Abbreviations

ANOVA: analysis of variance
CRWM: coronavirus-related WhatsApp message
HCW: health care worker
SNS: social networking sites
P: parameter
