# Supplementary material for: Demographic Factors Influencing the Impact of Coronavirus-Related Misinformation on WhatsApp: Cross-sectional Questionnaire Study
Source: JMIR Public Health Surveill. 2021 Jan 30;7(1):e19858. doi: 10.2196/19858 (PMC7850780; doi:10.2196/19858)
Supplement: Multimedia Appendix 1 [file publichealth_v7i1e19858_app1.pdf]

Study of WhatsApp as a source of information regarding Coronavirus

* Required

1. Language/ भाषा *

Mark only one oval.

- English *Skip to question 2*
- मराठी *Skip to question 29*

Personal Information

This study aims at analysing the outlook of the community towards WhatsApp as a source of information regarding Coronavirus.
DISCLAIMERS - Complete confidentiality will be maintained regarding all responses. This data will only be used for the analysis of the study stated above. The questions within this survey do not intend to hurt the sentiments of any political, religious organisation or community.

2. Age *

Mark only one oval.

- Below 18
- 19-25
- 26-35
- 36-50
- 51-65
- 66 and above

3. Gender *

Mark only one oval.

- Female
- Male
- Other: ______________________

4. City *

(Mention city of residence)

____

5. Occupation *

____

6. Do you use WhatsApp? *

Mark only one oval.

- Yes *Skip to question 7*
- No

Information regarding WhatsApp use

7. How much time do you spend on WhatsApp every day? *

Mark only one oval.

- 0-30 minutes
- 30 minutes - 1 hour
- 1 hour - 2 hours
- More than 2 hours

8. For what do you use WhatsApp? (Please rate as per priority) *

Mark only one oval per row.

	Low	Moderate	High
Messaging and chatting with friends and family			
Updates regarding work or college			
News and information			
Entertainment by photos, videos and forwarded messages			

9. How are you staying up to date with news regarding the coronavirus pandemic? (Select all that apply) *

Check all that apply.

- Newspapers
- Television news
- News on websites or apps (TOI, NDTV, CNN, e-Sakal, etc)
- WhatsApp
- Other social media (Facebook, Twitter, Instagram)
- Word of mouth
- Choosing not to stay updated

10. How often do you trust the information regarding Coronavirus you receive from the following? *

Mark only one oval per row.

	0%	25%	50%	75%	100%
Newspapers, television	◯	◯	◯	◯	◯
News Apps, News Websites	◯	◯	◯	◯	◯
WhatsApp	◯	◯	◯	◯	◯
Other Social Media platforms	◯	◯	◯	◯	◯

11. How often do you come across fake news on WhatsApp regarding Coronavirus? *

Mark only one oval.

- ◯ 0 times per day
- ◯ 1-3 times per day
- ◯ 4-6 times per day
- ◯ 7-9 times per day
- ◯ More than 10 times per day

12. Has any one of your close family members or friends been suspected or diagnosed with Coronavirus? *

Mark only one oval.

- ◯ Yes
- ◯ No

13. Which of the following helps you trust a WhatsApp message about Coronavirus? (Select a that apply) *

Check all that apply.

- [ ] An attached photo or video
- [ ] An attached link to a certain article or website
- [ ] When the source of the article has been mentioned
- [ ] Depends upon who sends the message
- [ ] None of the above

Skip to question 14

Look at the WhatsApp messages below and answer the questions accordingly. ***Please do not search online before answering***

DISCLAIMER - The authors of this survey neither accept nor refute the claims made ir these messages.

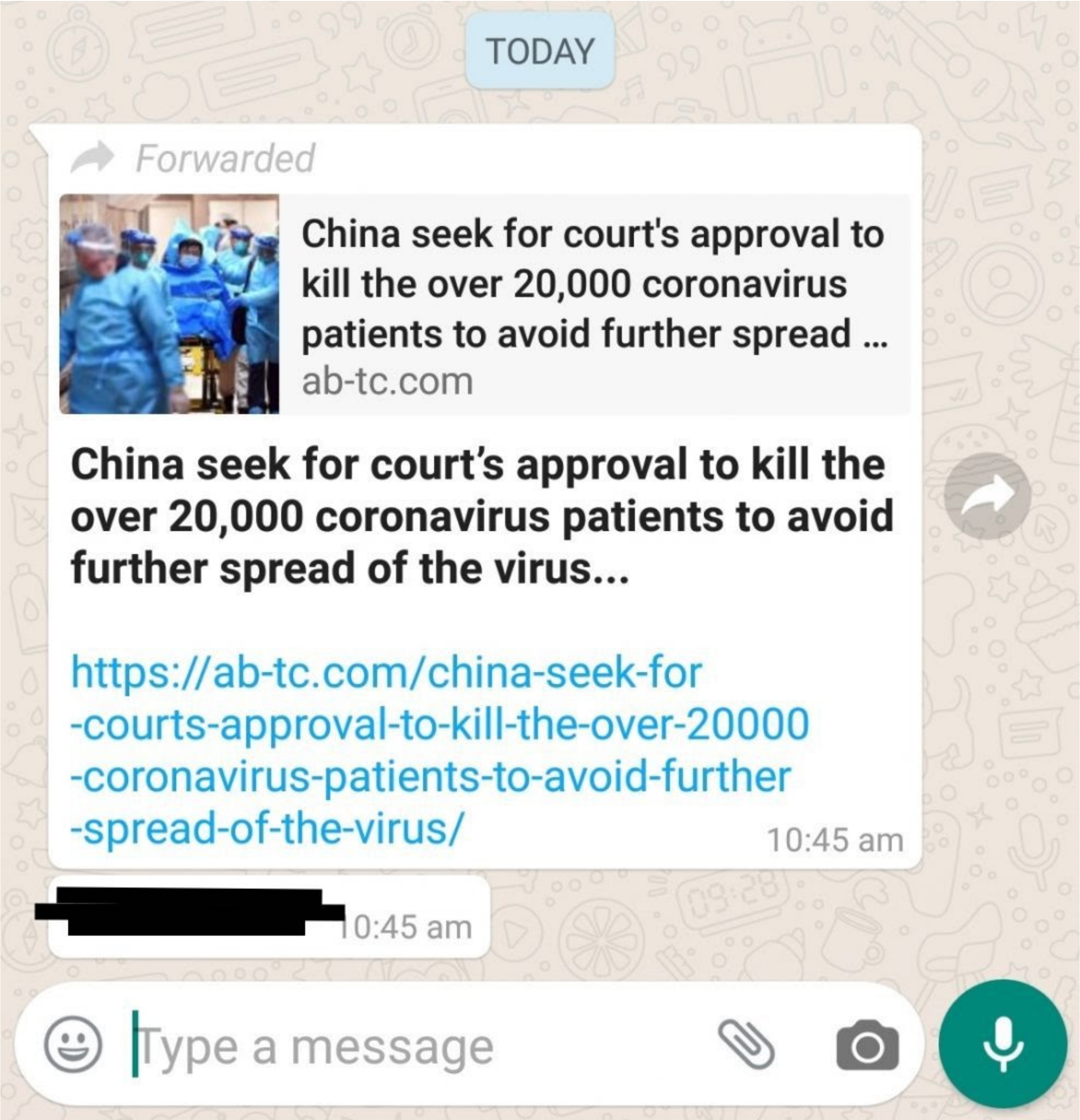
TODAY
Forwarded
China seek for court's approval to kill the over 20,000 coronavirus patients to avoid further spread ...
ab-tc.com
China seek for court's approval to kill the over 20,000 coronavirus patients to avoid further spread of the virus...
https://ab-tc.com/china-seek-for-courts-approval-to-kill-the-over-20000-coronavirus-patients-to-avoid-further-spread-of-the-virus/
10:45 am
[redacted] 10:45 am
Type a message

14. The above message is *

Mark only one oval.

- Definitely True
- Maybe True
- Maybe False
- Definitely False

Forwarded

Unicef

Corona virus is large in size where the cell diameter is 400-500 micro and for this reason **any mask prevents its entry**

The virus does not settle in the air but is grounded, so it is **not transmitted by air**.

Coronavirus when it falls on a metal surface, it will live 12 hours, so **washing hands with soap** and water well enough.

Corona virus when it falls on the fabric remains 9 hours, so **washing clothes** or **being exposed to the sun for two hours** meets the purpose of killing it.

The virus lives on the hands for 10 minutes, so putting an **alcohol sterilizer** in the pocket meets the purpose of prevention.

If the virus is exposed to a temperature of 26-27 ° C. it will be killed, as it does not live in hot regions. Also **drinking hot water and sun exposure** will do the trick

And **stay away from ice cream and eating cold** is important.

Gargle with warm and salt water kills the tonsils' germs and prevents them from leaking into the lungs.

Adherence to these instructions fulfills the purpose of preventing viruses.

UNICEF

08:22

15. The above message is *

Mark only one oval.

- ⬭ Definitely True
- ⬭ Maybe True
- ⬭ Maybe False
- ⬭ Definitely False

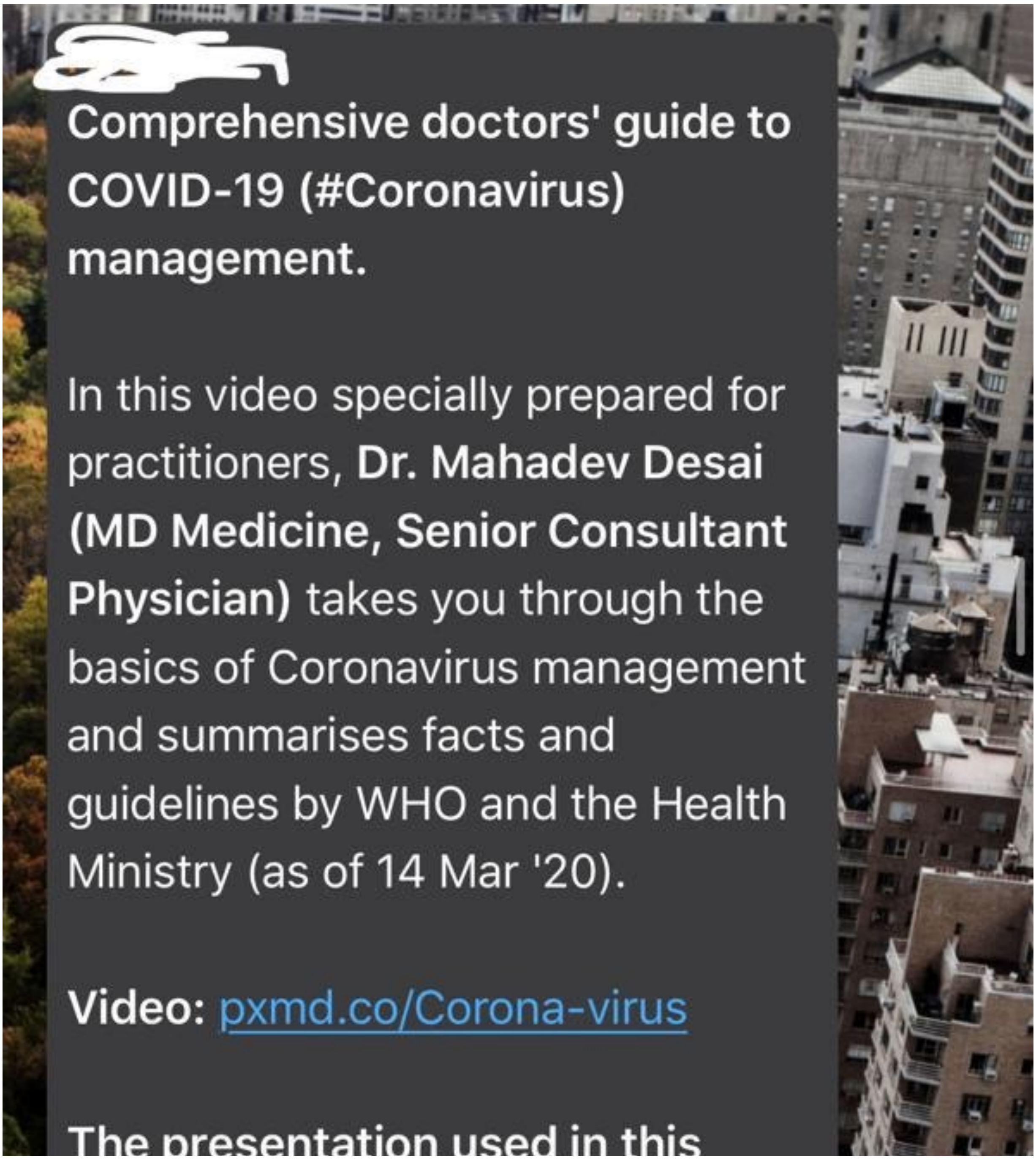

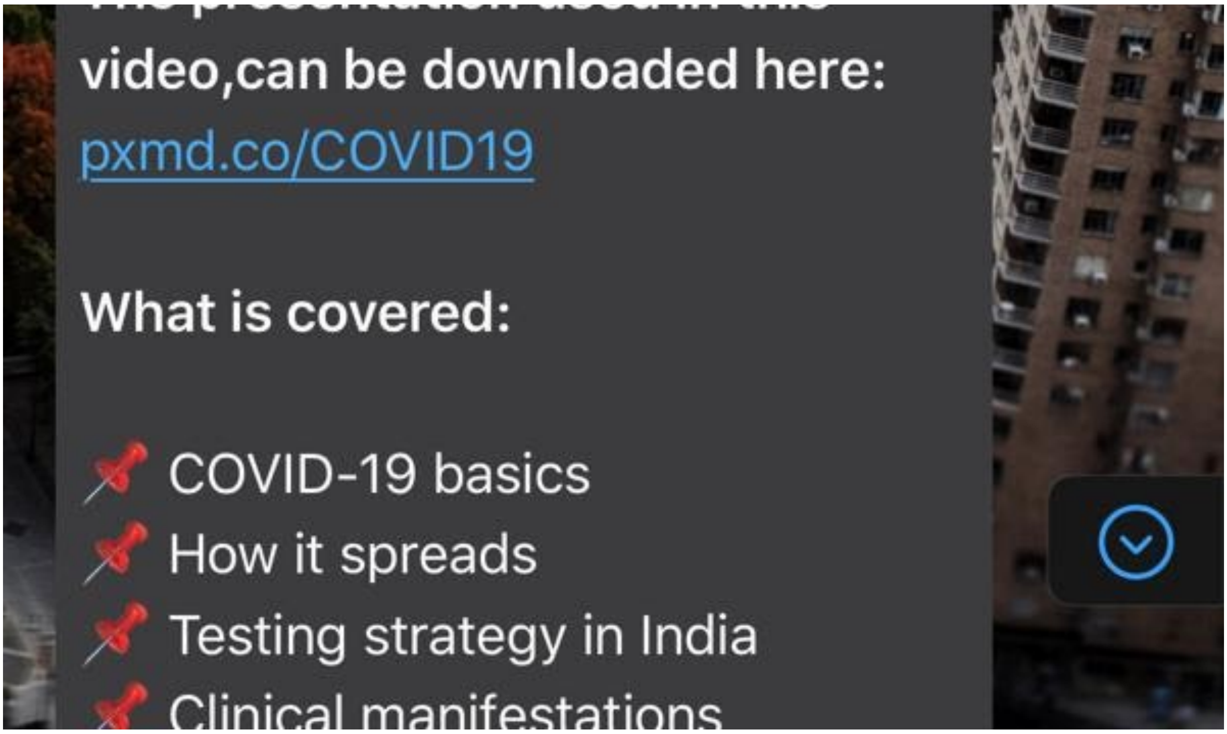

16. The above message is *

Mark only one oval.

- Definitely True
- Maybe True
- Maybe False
- Definitely False

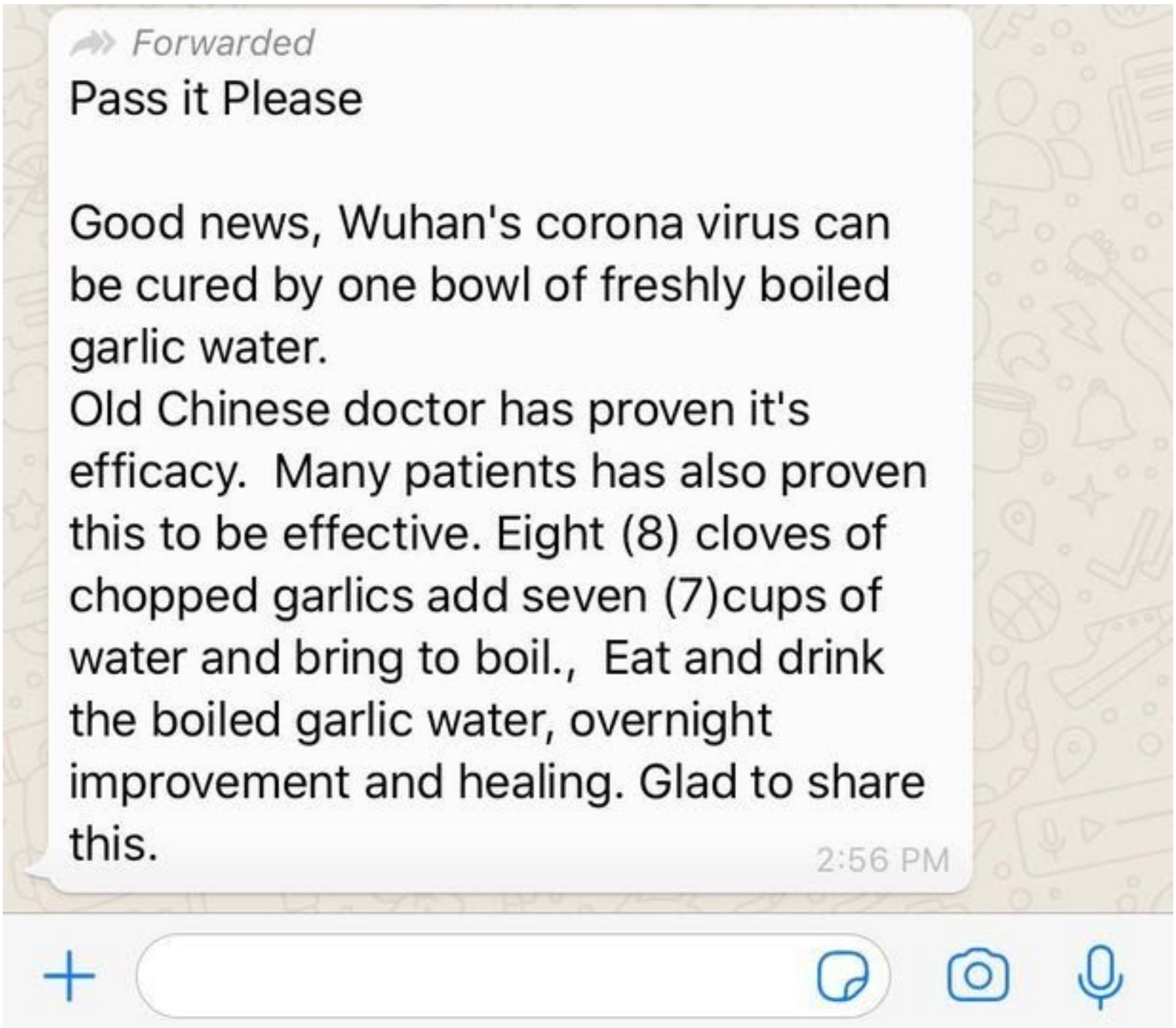

17. The above message is *

Mark only one oval.

- Definitely True
- Maybe True
- Maybe False
- Definitely False

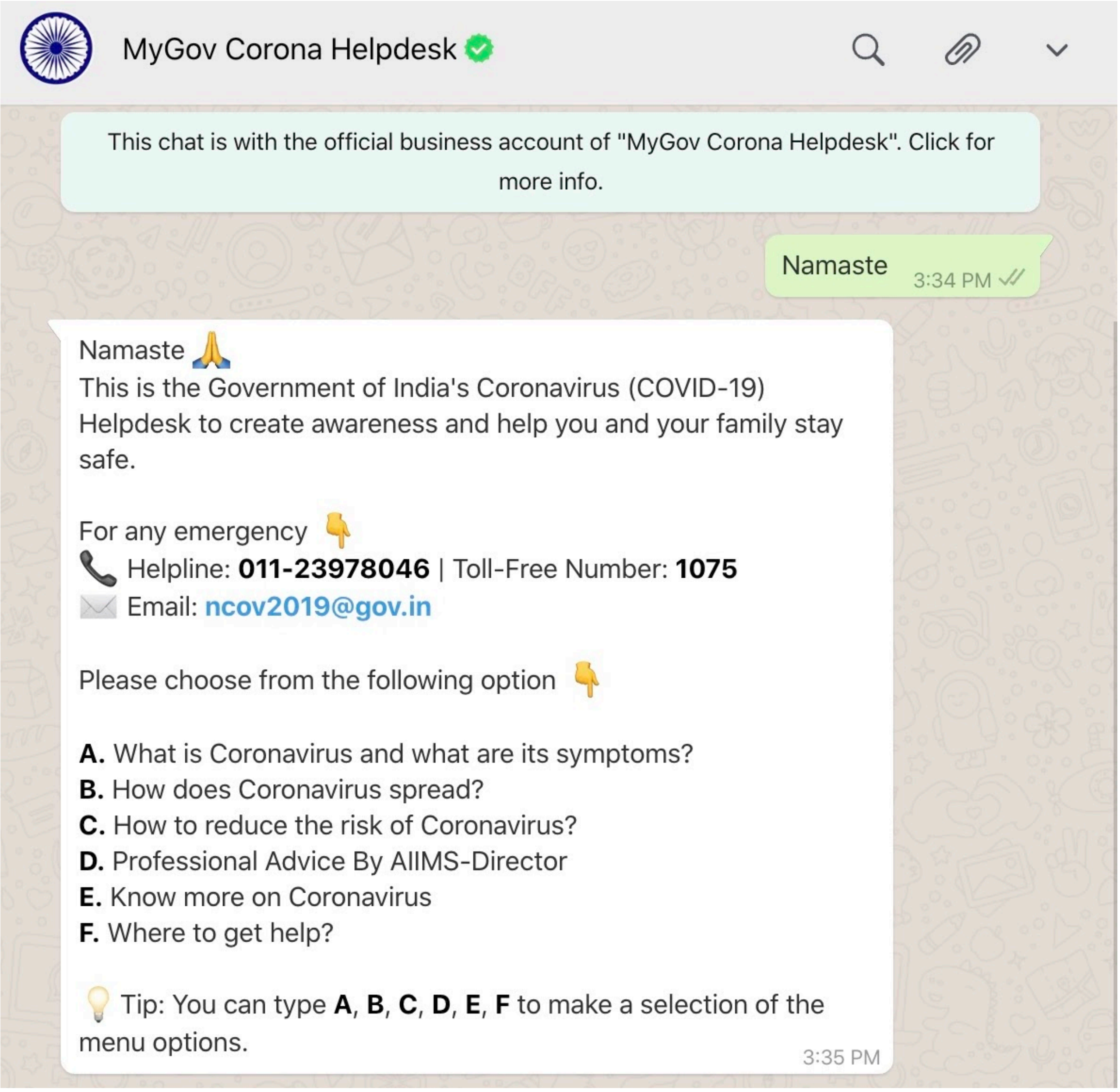

18. The above message is *

Mark only one oval.

- Definitely True
- Maybe True
- Maybe False
- Definitely False

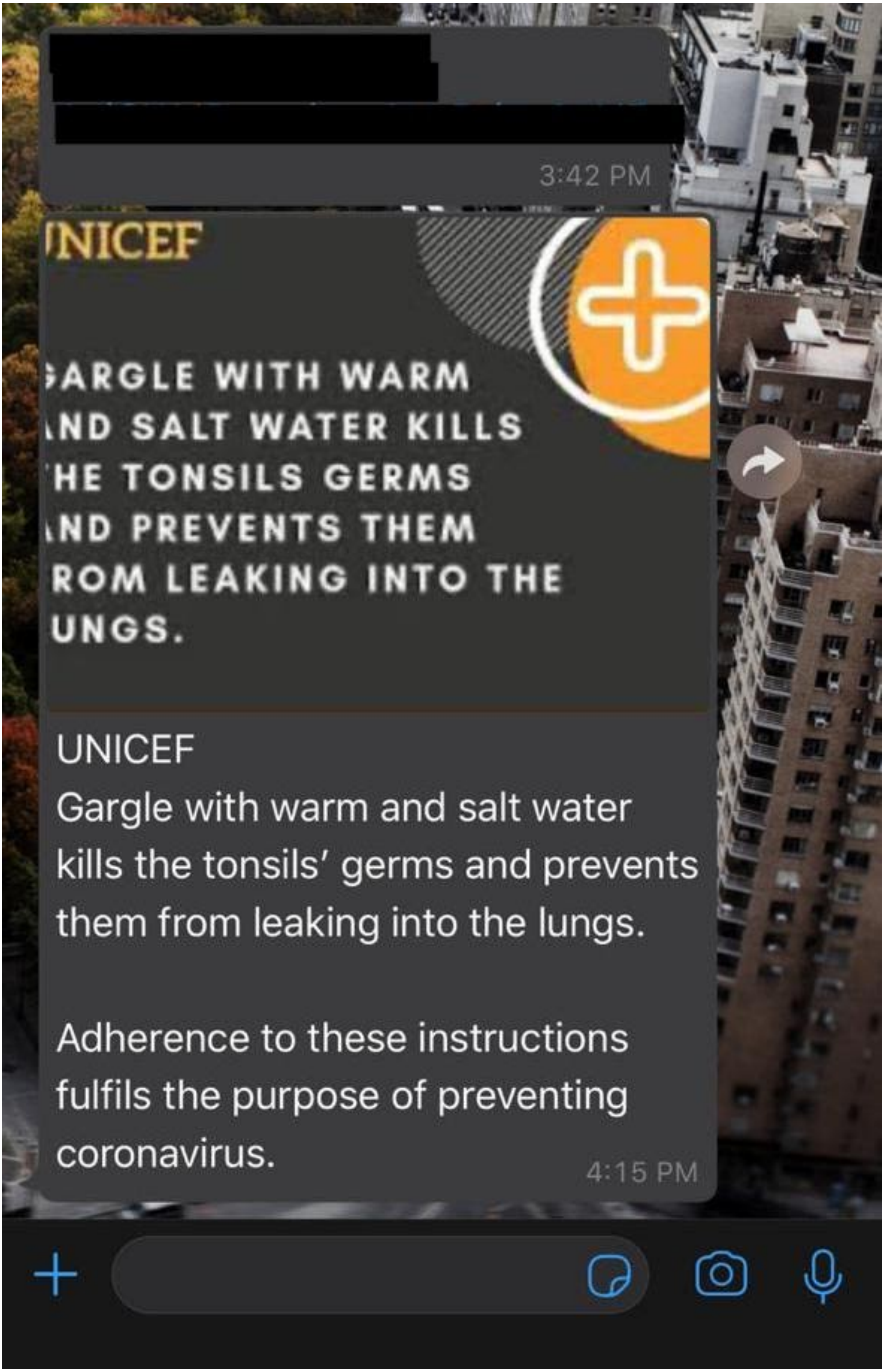
[redacted]
3:42 PM
NICEF
ARGLE WITH WARM
ND SALT WATER KILLS
HE TONSILS GERMS
ND PREVENTS THEM
ROM LEAKING INTO THE
UNGS.
UNICEF
Gargle with warm and salt water kills the tonsils' germs and prevents them from leaking into the lungs.
Adherence to these instructions fulfils the purpose of preventing coronavirus.
4:15 PM

19. The above message is *

Mark only one oval.

- Definitely True
- Maybe True
- Maybe False
- Definitely False

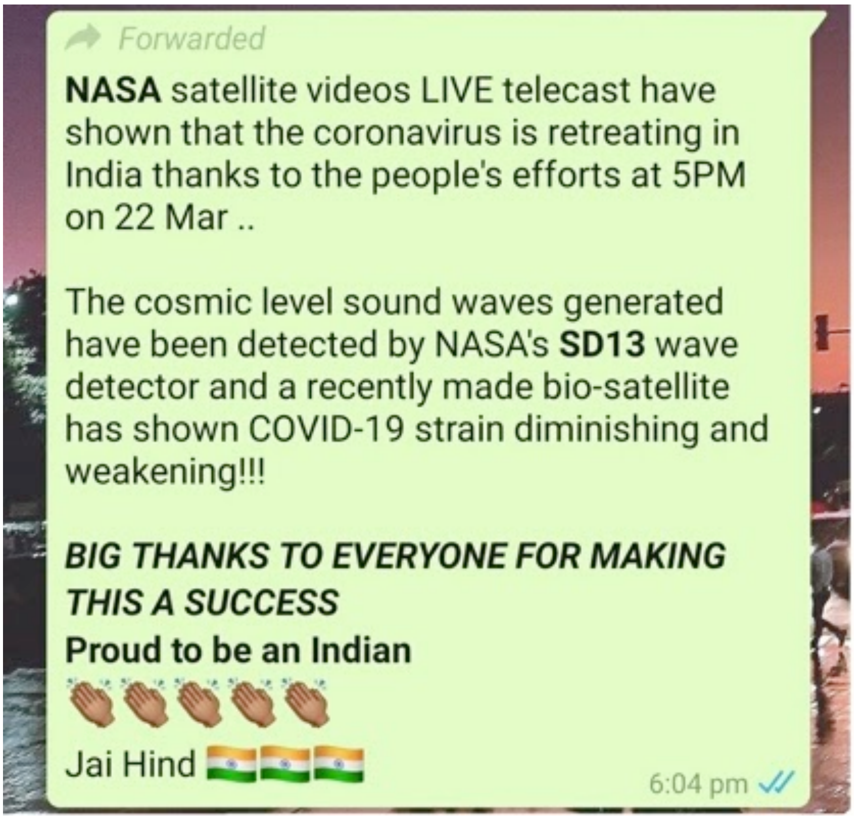

20. The above message is *

Mark only one oval.

- Definitely True
- Maybe True
- Maybe False
- Definitely False

Forwarded

HOMOEO MEDICINES FOR CORONA VIRUS DISEASE

Dear Friends,

There is lot of fear and anxiety among the people travelling abroad about the news about CORONA VIRUS or WUHAN VIRUS.

Do not worry about it. There are excellent medicines in Homoeopathy to prevent and also to cure. After studying the symptoms available through media our Forum strongly advise the following Homoeo medicines to prevent. We have 44 years of experience in handling various acute and chronic diseases though Homoeopathy. We provide this advise to help the needdy people.

ARSENIC ALB 30 daily morning 4 pills and evening 4pills for 5 days

No medicine on 6th day.

PHOSPHORUS 30 on 7th day morning 4 pills.

Daily use of the following Homoeo Mother Tinctures also will help to prevent and cure the disease. Use in following way for 15 days.

1. OCIMUM SANCTUM Mother Tincture (extract of Krishna Tulasi).
Drink morning 10 drops and evening 10 drops with 3 tea spoons of water.

2. TINOSPORA CORDIFOLIA Mother Tincture (extract of Amrita balli in Kannada and Thippa theega in Telugu).
Drink afternoon 10 drops and night 10 drops with 3 tea spoons of water.

Precautions:
1. Take all the precautions about cleanliness and hygiene.
2. Do not consume junk food and non-veg food.
3. Avoid Direct physical contact with other persons. Use nose mask.
4. Keep few Homoeo medicines and use when ever there is a need on the advise of Homoeopathic Doctor.
5. Kindly take medical help when there is a need. Do not neglect.

Our Forum wishes good health and happiness all. With regards and best wishes

P V Satyanarayana and all the Members of the Forum.
Dr. GV Chalapathi Memorial Homoeopathic Self Reliance Forum, (A Voluntary Service Organisation), Opposite to Scientists Hostel No 2, DRDO Township, C V Raman Nagar, Bangalore - 560093. Mobile No. 93430 94787

09:24

21. The above message is *

Mark only one oval.

- Definitely True
- Maybe True
- Maybe False
- Definitely False

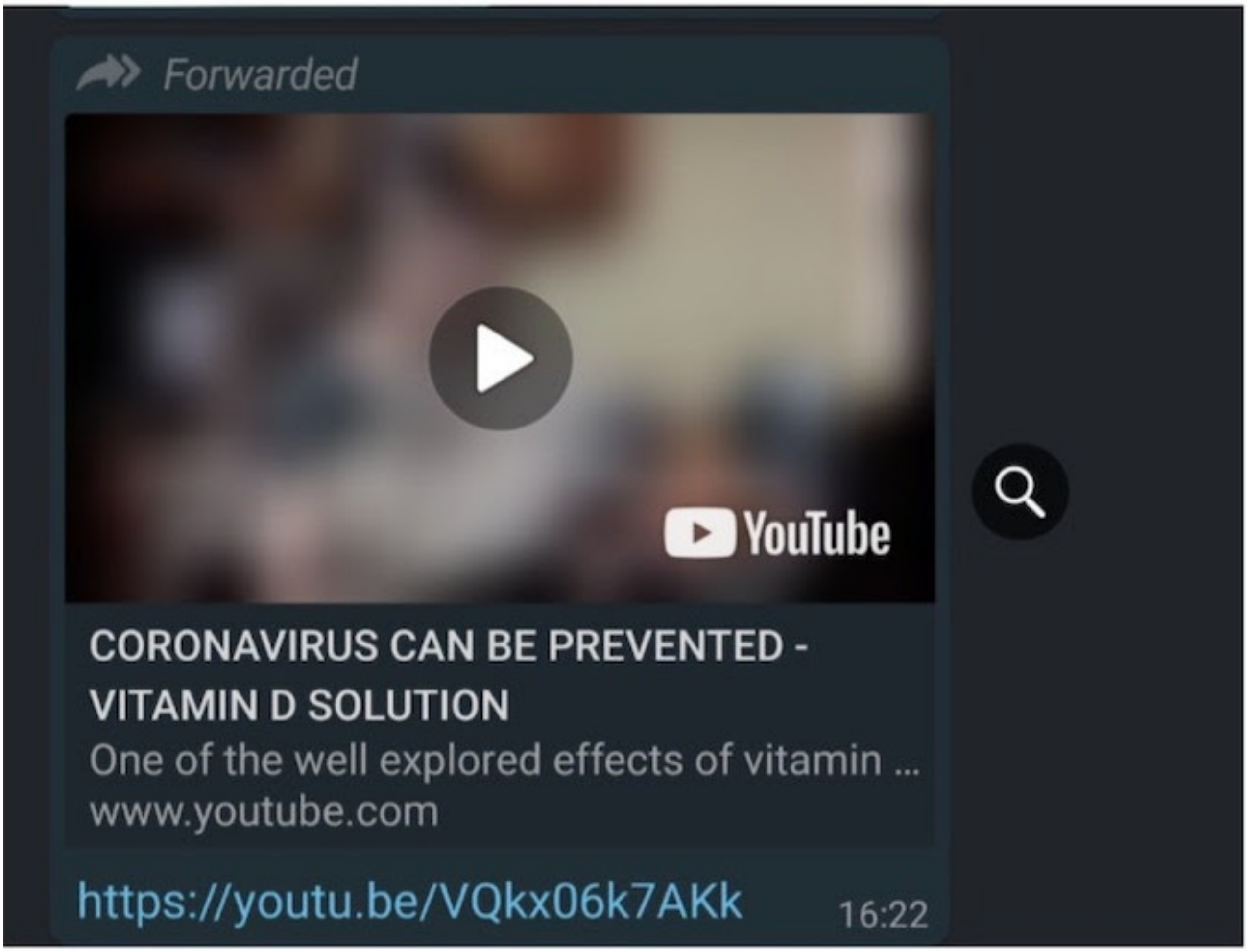

22. The above message is *

Mark only one oval.

- Definitely True
- Maybe True
- Maybe False
- Definitely False

[redacted]

Forwarded

WORLD HEALTH ORGANISATION PROTOCOL&PROCEDURE OF LOCKDOWN PERIODS FOR CONTROLLING ON MOST DANGEROUS VIRUS

STEP 1 - 1 DAY.
STEP 2- 21 DAYS.
AFTER 5 DAYS.
STEP 3- 28 DAYS.
AFTER 5 DAYS.
STEP 4 - 15 DAYS.

The sameway, our Indian governments are follow:
MAR22-1 DAY (TRIAL LOCKDOWN)

MAR24-APR14 - 21 DAYS(FIRST LOCKDOWN)

APRIL15- APRIL19 - RELAX FROM LOCKDOWN.

APR20 - MAY 18 - 28DAYS(SECOND LOCKDOWN)

INCASE,Covid19 patient ratio is Zero
Withdraw the LOCKDOWN.
Otherwise,
May19 - May 24 - Relax from LOCKDOWN.

May 25 - June 10 - 15 days (FINAL LOCKDOWN).

10:41 PM

23. The above message is *

Mark only one oval.

- Definitely True
- Maybe True
- Maybe False
- Definitely False

Skip to question 24

Information regarding WhatsApp use

24. How many messages about Coronavirus do you forward per day? *

Mark only one oval.

- 0-2
- 3-5
- 6-8
- More than 8

25. What percentage of messages about Coronavirus do you fact check before forwarding them? *

Mark only one oval.

- Never
- 1-25%
- 25-50%
- 50-75%
- 75-100%

26. Please provide your opinion regarding Coronavirus preventive measures commonly share on WhatsApp *

Mark only one oval per row.

	Never considered using	Considered but not used	Used once	Using regularly and recommending
Social Distancing	⬭	⬭	⬭	⬭
Masks	⬭	⬭	⬭	⬭
Allopathic Medicines	⬭	⬭	⬭	⬭
Herbal Medicines	⬭	⬭	⬭	⬭
Ayurvedic medicines	⬭	⬭	⬭	⬭
Homeopathic medicines	⬭	⬭	⬭	⬭
Home remedies (gaumutra, lime water with soda, rasam)	⬭	⬭	⬭	⬭

27. In your opinion, is WhatsApp a useful information tool during the current Coronavirus outbreak? *

Mark only one oval.

⬭ Yes

⬭ No

28. Any reasons for the above response?

कोरोनाव्हायरस संबंधी माहिती मिळवण्यासाठी व्हॉट्सॲपचा उपयोग ह्याचे विश्लेषण

कोरोनाव्हायरस संबंधी माहिती मिळवण्यासाठी व्हॉट्सॲपचा उपयोग करण्या मागील दृष्टिकोन तपासण्यासाठी आम्ही हे सर्वेक्षण व संशोधन करीत आहोत.
अस्वीकरण (डिस्क्लेमर) - आपल्या सर्वेक्षण उत्तरांबाबत संपूर्ण गोपनीयता पाळली जाईल. ही माहिती केवळ वर नमूद केलेल्या संशोधनाच्या विश्लेषणासाठी वापरली जाईल. या सर्वेक्षणातील कोणत्याही प्रश्नाचा हेतू कुठल्याही राजकीय किंवा धार्मिक संस्था अथवा संप्रदायाच्या भावना दुखावणे असे नाही.

*आवश्यक

वैयक्तिक माहिती

29. वय *

Mark only one oval.

- ( ) १८ किंवा कमी
- ( ) १९-२५
- ( ) २६-३५
- ( ) ३६ - ५०
- ( ) ५१ - ६५
- ( ) ६५ च्या वर

30. लिंग *

Mark only one oval.

- ( ) स्त्री
- ( ) पुरुष
- ( ) इतर

31. शहर *

राहण्याचे शहर लिहावे

____

32. व्यवसाय *

____

33. आपण व्हॉट्सॲप वापरता का? *

Mark only one oval.

- ( ) होय *Skip to question 34*
- ( ) नाही

Skip to question 34

व्हॉट्सॲप वापर संबंधित माहिती

34. आपण दररोज व्हॉट्सॲप किती वेळ वापरता? *

Mark only one oval.

- ( ) ० - ३० मिनिटे
- ( ) ३० मिनिटे - १ तास
- ( ) १ तास - २ तास
- ( ) २ तासांपेक्षा जास्त

35. आपण व्हॉट्सॲप कशासाठी वापरता? (कृपया महत्वाप्रमाणे निवडा) *

Mark only one oval per row.

	कमी	मध्यम	जास्ती
मित्र/ मैत्रीण आणि कुटुंबियांसह गप्पा मारण्यासाठी	◯	◯	◯
कामासंबंधित किंवा महाविद्यालयासंबंधित माहितीसाठी	◯	◯	◯
बातम्या आणि माहितीसाठी	◯	◯	◯
फोटो, व्हिडिओ आणि फॉर्वर्डेड मेसेजीसद्वारे मनोरंजनासाठी	◯	◯	◯

36. कोरोनाव्हायरस संबंधित ताज्या बातम्या आपल्याला कुठून कुठून मिळतात? (लागू असणाऱ्या सर्व गोष्टी निवडा) *

Check all that apply.

- [ ] वर्तमानपत्रे
- [ ] TV वरील बातम्या
- [ ] वेबसाइट्स किंवा ॲप्सवरील बातम्या (टीओआय, एनडीटीव्ही, सीएनएन, ई- सकाळ , इ.)
- [ ] व्हॉट्सॲप
- [ ] इतर सोशल मीडिया (फेसबुक, ट्विटर, इंस्टाग्राम, इ.)
- [ ] नातेवाईकांकडून व शेजार पाजारच्या लोकांकडून
- [ ] ताज्या बातम्यांच्या संपर्कात नाही

37. कोरोनाव्हायरस संबंधित माहिती मिळवण्यासाठी खालीलपैकी माध्यमांवर आपला किती टक्के विश्वास आहे? *

Mark only one oval per row.

	०%	२५%	५०%	७५%	१००%
वर्तमानपत्रे, दूरदर्शन, रेडिओ	◯	◯	◯	◯	◯
वेबसाइट्स किंवा ॲप्सवरील बातम्या	◯	◯	◯	◯	◯
व्हॉट्सॲप	◯	◯	◯	◯	◯
इतर सोशल मीडिया (फेसबुक, ट्विटर, इंस्टाग्राम)	◯	◯	◯	◯	◯

38. कोरोनाव्हायरस संदर्भात व्हॉट्सॲपवर आपल्याला दिवसातून किती वेळा खोट्या बातम्या येतात? *

Mark only one oval.

- ० वेळा
- १-३ वेळा
- ४-६ वेळा
- ७-९ वेळा
- १० पेक्षा जास्तं

39. आपल्या कुटुंबातील कोणत्याही सदस्याला किंवा जवळच्या मित्र /मैत्रिणीला कोरोनाव्हायरसच्या आजाराचा संशय किं निदान झाले आहे का? *

Mark only one oval.

- होय
- नाही

40. कोरोनाव्हायरसबद्दल व्हॉट्सॲपवर आलेल्या मेसेजवर विश्वास ठेवताना खालीलपैकी आपण कोणत्या गोष्टी विचारात घेता? (लागू असणाऱ्या सर्व गोष्टी निवडा) *

Check all that apply.

- सोबत जोडलेला फोटो किंवा व्हिडिओ
- वेबसाईट किंवा माहितीपत्राची लिंक
- मेसेजच्या लेखकाचा किव्हा उगमाचा उल्लेख
- कोण मेसेज पाठवते त्यावर अवलंबून
- वरील पैकी काहीच नाही

Skip to question 41

खालील व्हॉट्सॲपचे मेसेजेस पहा आणि त्यानुसार प्रश्नांची उत्तरे द्या. * कृपया उत्तर देताना ऑनलाईन शोधू नका *****

अस्वीकरण - या सर्वेक्षणाचे लेखक या मेसेजेस मध्ये केलेले दावे स्वीकारत नाहीत किंवा नाकारत नाहीत.

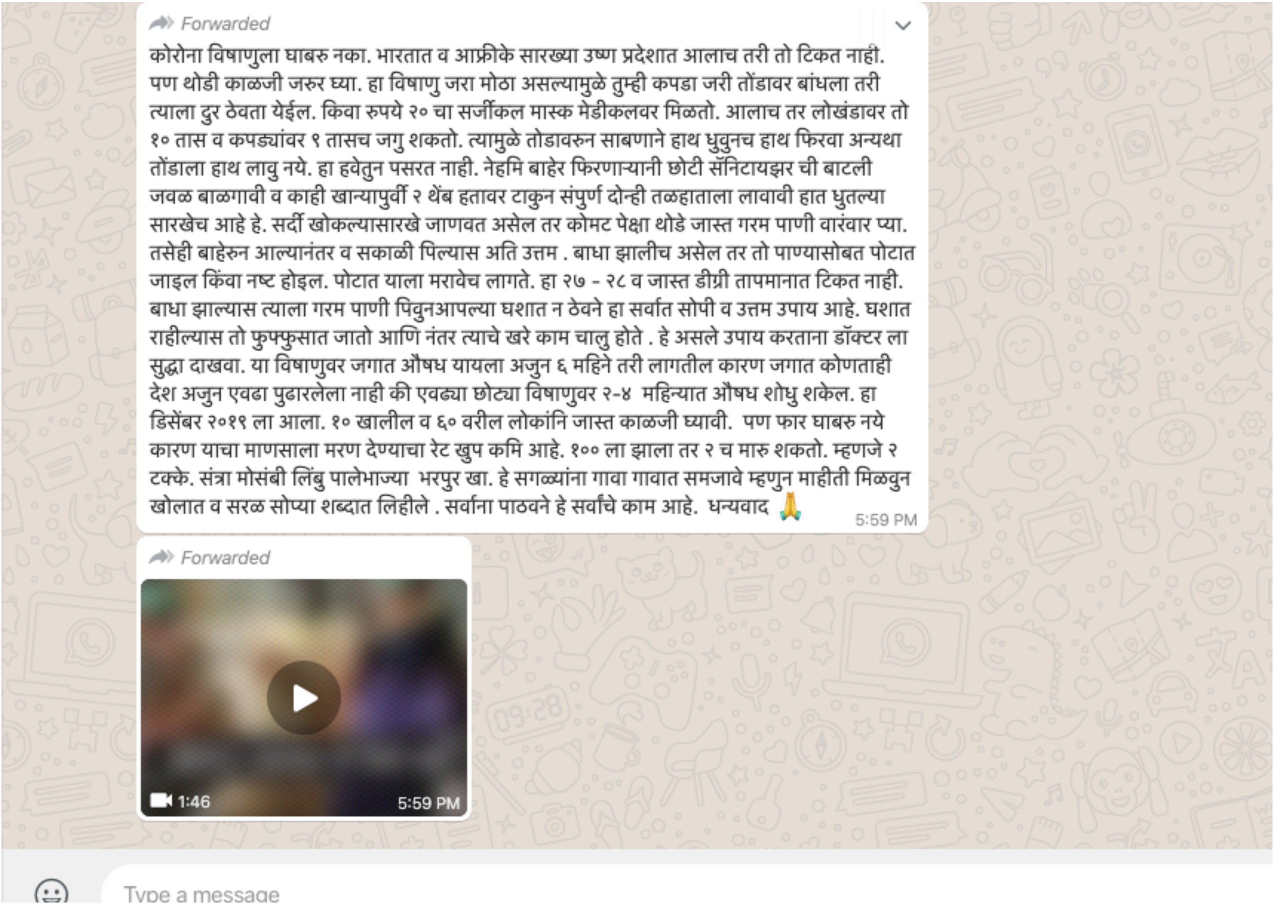

41. वरील मेसेज *

Mark only one oval.

- ( ) निश्चितपणे सत्य आहे
- ( ) कदाचित सत्य असेल
- ( ) कदाचित खोटे असेल
- ( ) निश्चितपणे खोटे आहे

Forwarded

एक मेणबत्ती 2KCal उष्णता देते

एक मोबाईल flash 0.5kcal उष्णता देतो.

एक तेलाचा दिवा 3kcal उष्णता देतो.

समजा 130 करोड लोकांमध्ये
70 करोड लोकांनी हा आदेश पाळला

आणि त्यात 35 करोड मेणबत्ती, 20 करोड flash

आणि 15 करोड दिवे पेटवले गेले,

तर 125 करोड kcal उष्णता निर्माण होईल.

कोरोना 10 kcal उष्णतेत मरून जातो.

त्यामुळे 5 एप्रिल ला सर्व कोरोना विषाणू मरून जाणार.

मोदीजींचा हा मास्टरस्ट्रोक आहे (5 एप्रिल रात्री 9 वाजता नक्की दिवे लावा वेळेवर). रात्री 9:30 नंतर आपला देश कोरोना मुक्त असेल. पुढचे आठ दहा दिवसात कदाचित संपूर्ण देशात रोगप्रतिकारक औषधांने फवारणी केली जाईल. 15 एप्रिल ला देश पुन्हा एकदा महासत्तेकडे वाटचालीला सुरवात करेल.

भारतातील प्रत्येक नागरीकापर्यंत हा मेसेज पोहोचवा.

महत्त्वाची सूचना : आपण दिवे लावणार असाल तर त्यात कापूर टाकायला विसरू नका. त्यामुळे बाकीचे रोगजंतू पण मरून जातील.

1:16 PM

42. वरील मेसेज *

Mark only one oval.

- निश्चितपणे सत्य आहे
- कदाचित सत्य असेल
- कदाचित खोटे असेल
- निश्चितपणे खोटे आहे

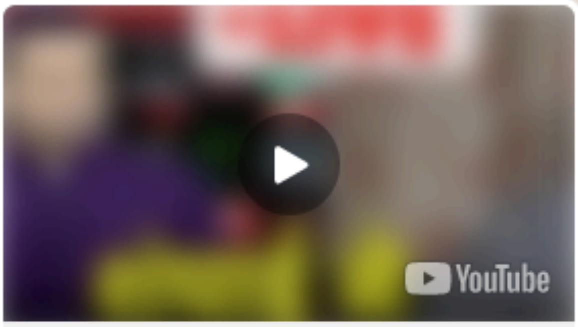

China में लाखों जानें बचाने वाले Dr Sanjeev कहते हैं India में अभी और ज़ोर पकड़ेगा Coronavirus

News24 motto of 'Think First' is reflected in its

www.youtube.com

ज्यांचे वय वर्ष १० ते ५० यामध्ये आहे व ज्यांना कोणताही इतर आजार नाही तसेच ज्यांचे आरोग्य ठीक आहे, त्यांच्या शरीरात हा विषाणू गेल्यानंतर त्या विषाणू बरोबर आपले शरीर ७ ते ८ दिवस युध्द करते आणि ७ ते ८ दिवसात कोरोनो व्हायरसला मारून टाकते,
चीन मधील भारतीय डॉक्टर Dr. Sanjiv Chube ज्यांनी स्वःता 30 हजार ते 40 हजार कोरोना व्हायरस बाधीत व्यक्तिवर उपचार केले ते सांगतात , यावर काही औषध जरी नाही घेतले तरी तुमचे शरीर ७ ते ८ दिवसामधे या कोरोना व्हायरसला मारून टाकते, ते असे पण सांगतात 85% रूग्ण हे घरी राहून बरी झाली .
आम्ही त्यांना Antiviral medicine दिली.
Antiviral medicine म्हणजे विशेष काही नाही......
आपल्या भारतात मिळणारी VICKS ACTION 500 ही एक Antiviral Medicine आहे ती तुम्ही वापरू शकता. तुम्ही पूर्णपणे बरे होशाल........ असे डॉ Sanjiv Chaube सांगतात.

त्यामुळे या आजाराला घाबरू नका, घरी रहा, सकस आहार घ्या, व्यायाम करा आणि तुमचे आई वडील, भाऊ बहिण , मित्र व नातेवाईक यांना व्यवस्थित समजून सांगा कारण त्यांची भीती व गैरसजूत घालवणे आपले कर्तव्य आणि जवाबदारी आहे.
चीन मधील डॉक्टर संजीव चौबे यांच्या मुलाखतीची लिंक दिली आहे.
©गणेश गोर्डे.
https://youtu.be/ksPQvdRfJVI

2:57 PM

Type a message

43. वरील मेसेज *

Mark only one oval.

- ◯ निश्चितपणे सत्य आहे
- ◯ कदाचित सत्य असेल
- ◯ कदाचित खोटे असेल
- ◯ निश्चितपणे खोटे

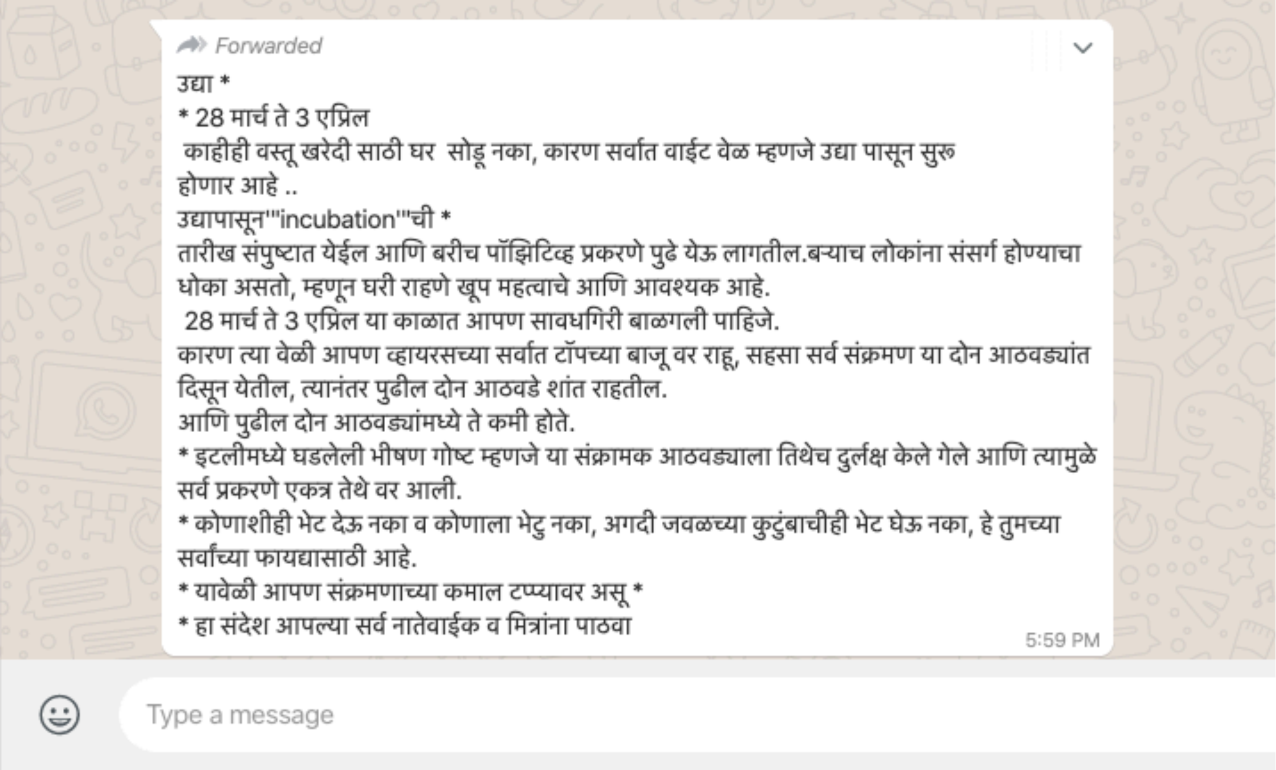

44. वरील मेसेज *

Mark only one oval.

- ◯ निश्चितपणे सत्य आहे
- ◯ कदाचित सत्य असेल
- ◯ कदाचित खोटे असेल
- ◯ निश्चितपणे खोटे

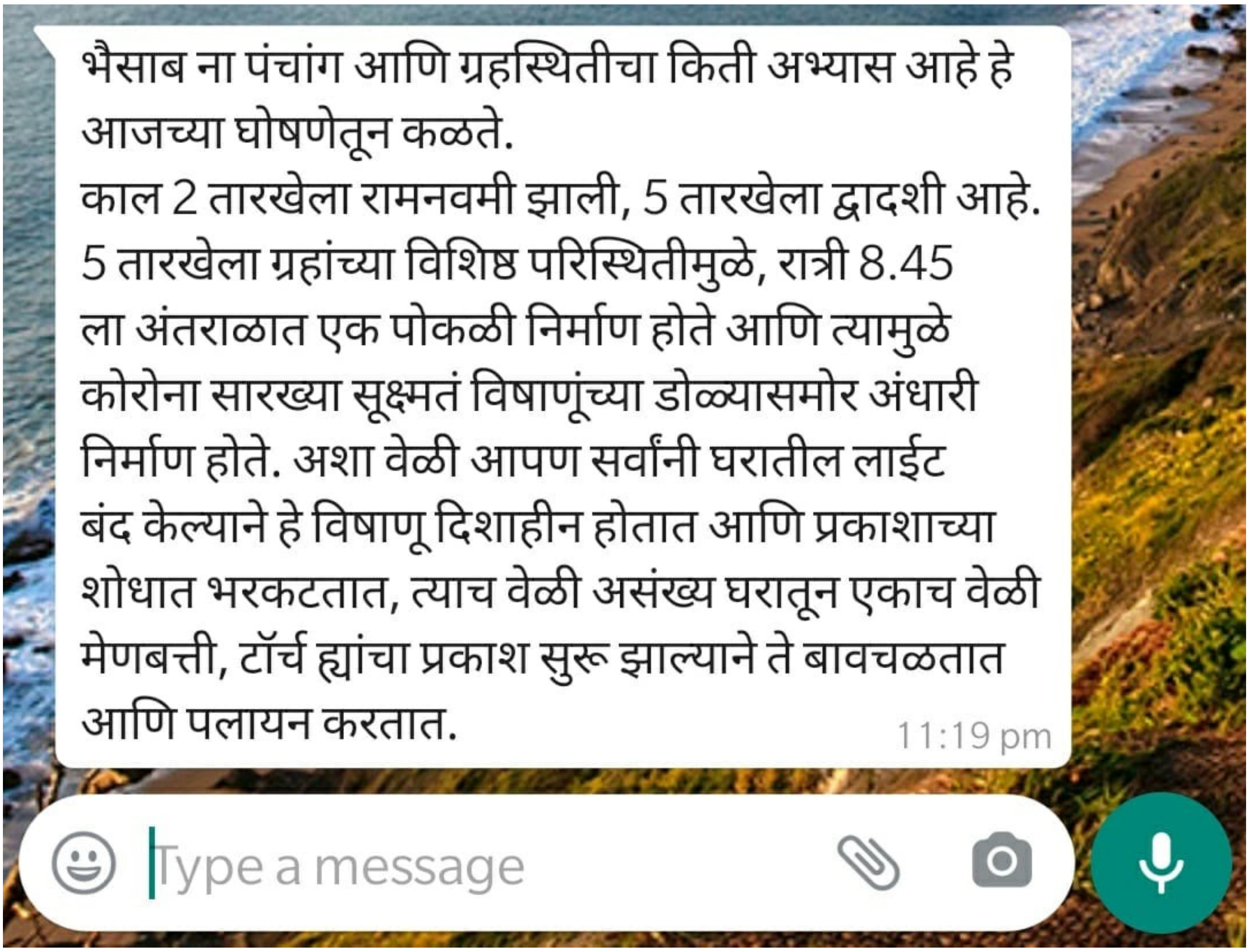

45. वरील मेसेज *

Mark only one oval.

- निश्चितपणे सत्य आहे
- कदाचित सत्य असेल
- कदाचित खोटे असेल
- निश्चितपणे खोटे आहे

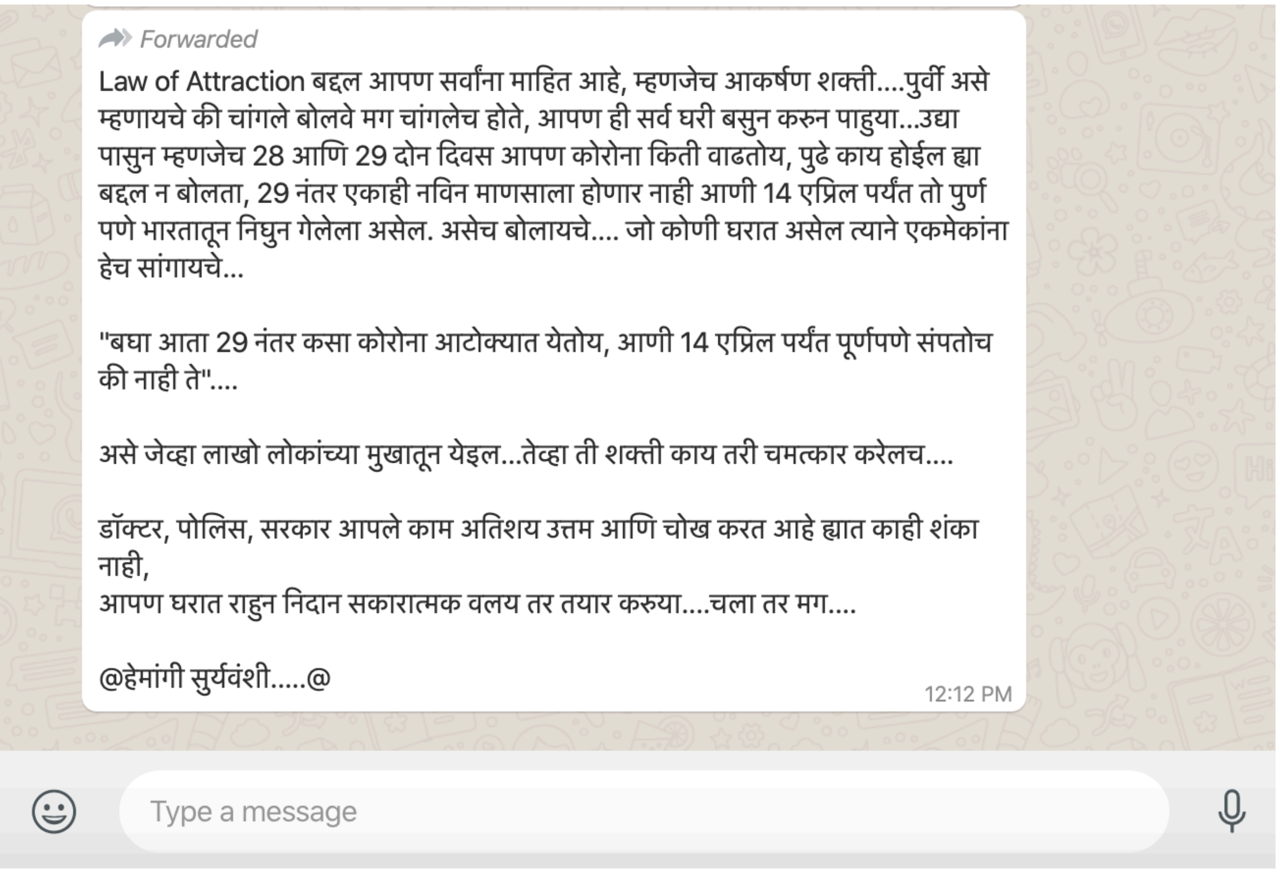

46. वरील मेसेज *

Mark only one oval.

- निश्चितपणे सत्य आहे
- कदाचित सत्य असेल
- कदाचित खोटे असेल
- निश्चितपणे खोटे आहे

५ दिनांक, ९ वाजता,
९ मिनिटेच का?
तुम्हाला पतंग माहिती आहे का? तोच तो जो पेटत्या दिव्यावर आकर्षित होऊन झडप घालतो आणि स्वत:च जळून खाक होतो.
आपल्या पुराणात ९ अंकाचे किती महत्व आहे हे वेगळं सांगायची गरज नाही, आता बघा ९ ग्रह, नवरात्रीचे ९ दिवस, ९ अवतार एव्हढच काय तर मुल जन्माला घालायचा कालावधी पण ९ महिने हा आहे.
मोदींनी ९ ची वेळ आणि ९ मिनिटेच का निवडली हे एव्हाना तुमच्या लक्षात आलेच असेल, काही लोकांना शंका ही आहे की ५ एप्रिल ही तारीख का निवडली? ९ का नाही, तर त्यामागे पण एक लॉजिक आहे, ५+४(चौथा महिना)= ९. मोदीजी जे काही करतात ते सर्व विचार करूनच करतात.
आता वर पतंगाचे उदाहरण का दिले हा तुम्हाला प्रश्न पडला असेल, त्यासाठी एक उदाहरण सांगतो, १८९३ साली आजच्या कोरोना सारखीच एका विषाणूची साथ जगभर आली होती, त्यावेळी पूर्ण जगात हाहाकार उडाला होता, त्यावेळी जगभरातील लोकसंख्येच्या जवळपास १२% लोक त्या साथीत मृत्युमुखी पडले होते, पण आश्चर्य म्हणजे भारतात त्या साथीचा एकही बळी गेला नव्हता, सर्व जगभर याबद्दल आश्चर्य व्यक्त केले गेले.
त्यावेळी इंग्लडमधील एक शास्त्रज्ञ जॉन विल्यम्सन हा खास याबद्दल माहिती घेण्यासाठी भारतात आला, त्याने दोन महिने पूर्ण अभ्यासा अंती हे मत मांडले की, जगभर विषाणूची साथ असताना भारतात एकही बळी गेला नाही, कारण त्यावेळी दिवाळी होती, आणि दिवाळी असल्याने सर्व भारत दिव्यांनी उजळून गेला होता, त्याच्या हे लक्षात आले की दिवा लावल्याने ज्याप्रमाणे पतंग दिव्यावर झोकून देतो त्याच प्रमाणे विषाणू पण दिव्यावर झोकून देतात आणि त्याचा नायनाट होतो, पतंग मोठा असल्याने आपल्याला डोळ्याने दिसतो, विषाणू दिसत नसल्याने आपल्या हे लक्षात येत नाही,
अजून दुसरी एक गोष्ट १९३४ साली संस्कृत पंडित भार्गव आचार्य यांनी आपल्या प्रवचनात सांगितली होती, विषाणू हे नाव विष्णू वरून पडले असावे, लक्ष्मी च्या विरहात सूक्ष्म रुपात सैरभर फिरणारा विष्णू चा अणू म्हणजे विषाणू आणि आपल्याला माहितीच आहे, संध्याकाळी दिवे लावल्यावर विष्णू पत्नी लक्ष्मी ही दिव्यारूपी त्या घरात प्रवेश करते, आणि साहजिकच आहे विष्णू लक्ष्मी कडे ओढला जाणार,
मित्रांनो, मोदी जे काही करतात ते पूर्ण अभ्यास करूनच करतात हे आपल्या सर्वांनाच माहिती आहे, या गोष्टींची खात्री करण्याची जबाबदारी त्यांनी इस्रो चे शास्त्रज्ञ व्यंकय्याधरण यांच्यावर सोपवली होती, ते स्वतः संस्कृतपंडित आहेत, त्यांनी यावर पूर्ण अभ्यास करून ही उपाययोजना मोदींच्या समोर मांडली, मोदींनी क्षणाचा विलंब न करता, ही अमलात आणायचे धोरण आखले,
माझी सर्वांना विनंती आहे की दिवा पेटवल्यावर, शुभंकरोती कल्याणम म्हणायला विसरू नका, विचार करा हा मंत्र १३० कोटी जनतेने एकत्रित म्हणला तर आसमंतात नवीन चैतन्य निर्माण होऊन आपल्यात ऊर्जा संचारेल.
काही लोकांना वाटतं की मोदी काय करत आहेत? ते गप्प का? पण अहोरात्र झटणारा पंतप्रधान आपल्याला लाभला आहे हेच आपलं भाग्य आहे, आपण सर्वांनी मोदींना साथ द्या.
नमो नमो
#लढा_कोरोनासी

11:32 AM

Type a message

47. वरील मेसेज *

Mark only one oval.

- निश्चितपणे सत्य आहे
- कदाचित सत्य असेल
- कदाचित खोटे असेल
- निश्चितपणे खोटे आहे

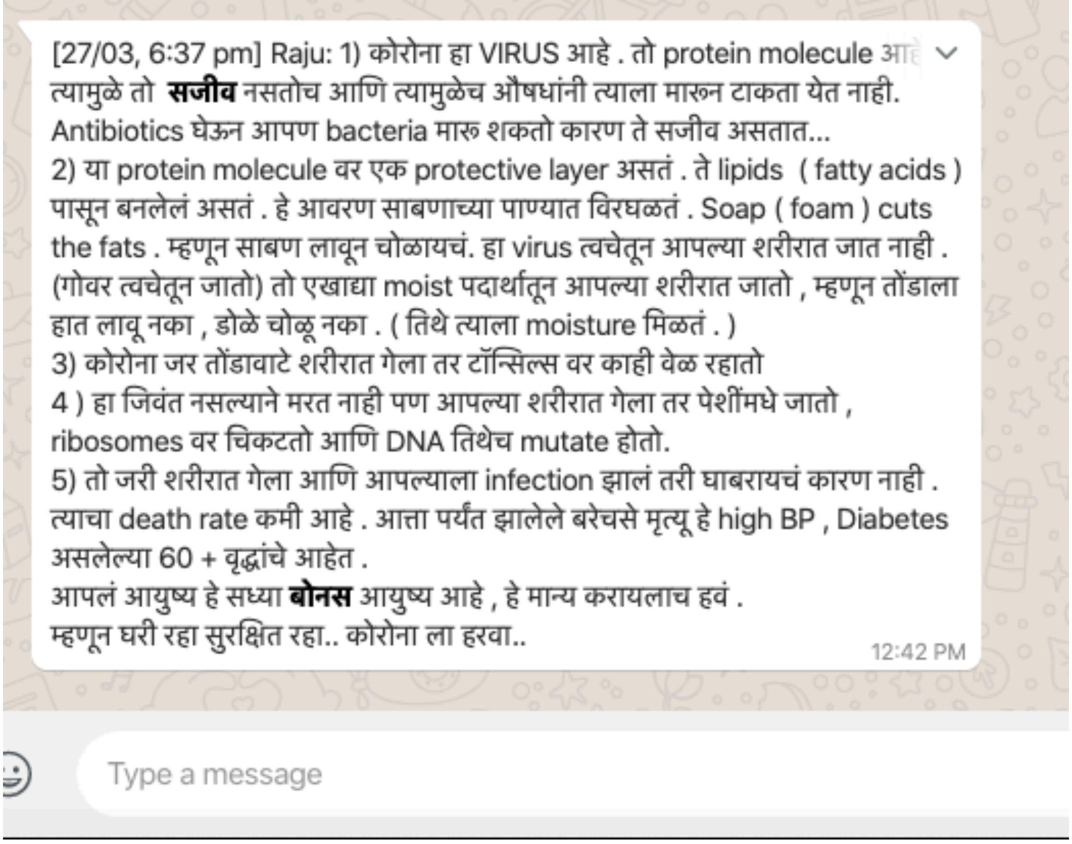

[27/03, 6:37 pm] Raju: 1) कोरोना हा VIRUS आहे . तो protein molecule आहे
त्यामुळे तो **सजीव** नसतोच आणि त्यामुळेच औषधांनी त्याला मारून टाकता येत नाही.
Antibiotics घेऊन आपण bacteria मारू शकतो कारण ते सजीव असतात...
2) या protein molecule वर एक protective layer असतं . ते lipids (fatty acids)
पासून बनलेलं असतं . हे आवरण साबणाच्या पाण्यात विरघळतं . Soap (foam) cuts
the fats . म्हणून साबण लावून चोळायचं. हा virus त्वचेतून आपल्या शरीरात जात नाही .
(गोवर त्वचेतून जातो) तो एखाद्या moist पदार्थातून आपल्या शरीरात जातो , म्हणून तोंडाला
हात लावू नका , डोळे चोळू नका . (तिथे त्याला moisture मिळतं .)
3) कोरोना जर तोंडावाटे शरीरात गेला तर टॉन्सिल्स वर काही वेळ रहातो
4) हा जिवंत नसल्याने मरत नाही पण आपल्या शरीरात गेला तर पेशींमधे जातो ,
ribosomes वर चिकटतो आणि DNA तिथेच mutate होतो.
5) तो जरी शरीरात गेला आणि आपल्याला infection झालं तरी घाबरायचं कारण नाही .
त्याचा death rate कमी आहे . आत्ता पर्यंत झालेले बरेचसे मृत्यू हे high BP , Diabetes
असलेल्या 60 + वृद्धांचे आहेत .
आपलं आयुष्य हे सध्या **बोनस** आयुष्य आहे , हे मान्य करायलाच हवं .
म्हणून घरी रहा सुरक्षित रहा.. कोरोना ला हरवा..
12:42 PM

Type a message

48. वरील मेसेज *

Mark only one oval.

- निश्चितपणे सत्य आहे
- कदाचित सत्य असेल
- कदाचित खोटे असेल
- निश्चितपणे खोटे आहे

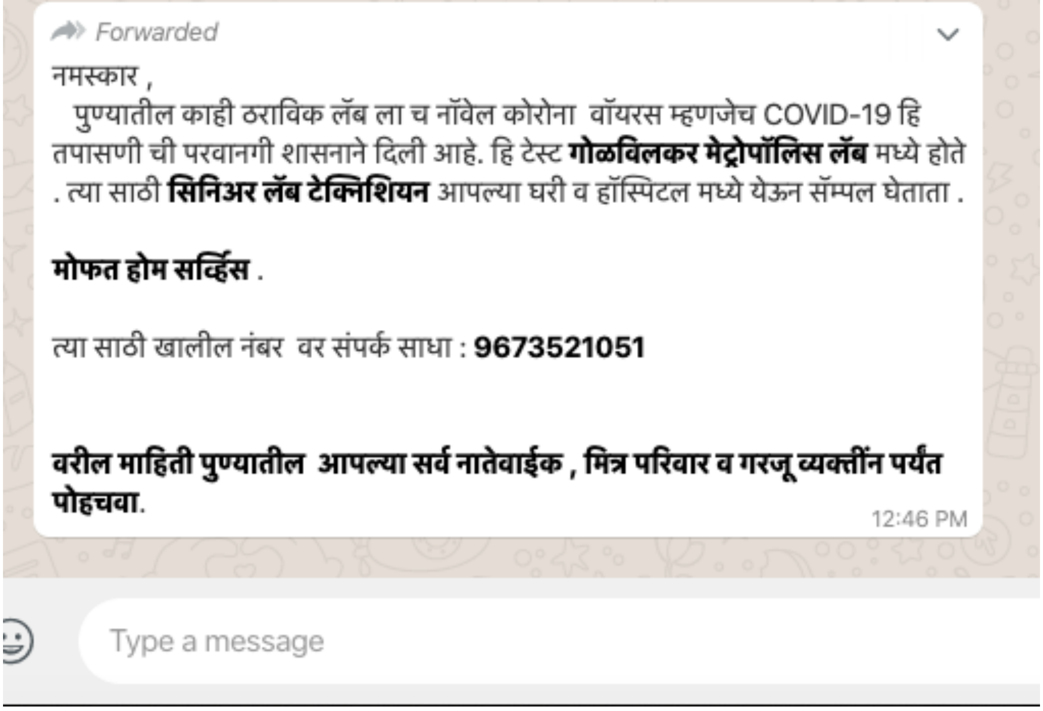

49. वरील मेसेज *

Mark only one oval.

- निश्चितपणे सत्य आहे
- कदाचित सत्य असेल
- कदाचित खोटे असेल
- निश्चितपणे खोटे आहे

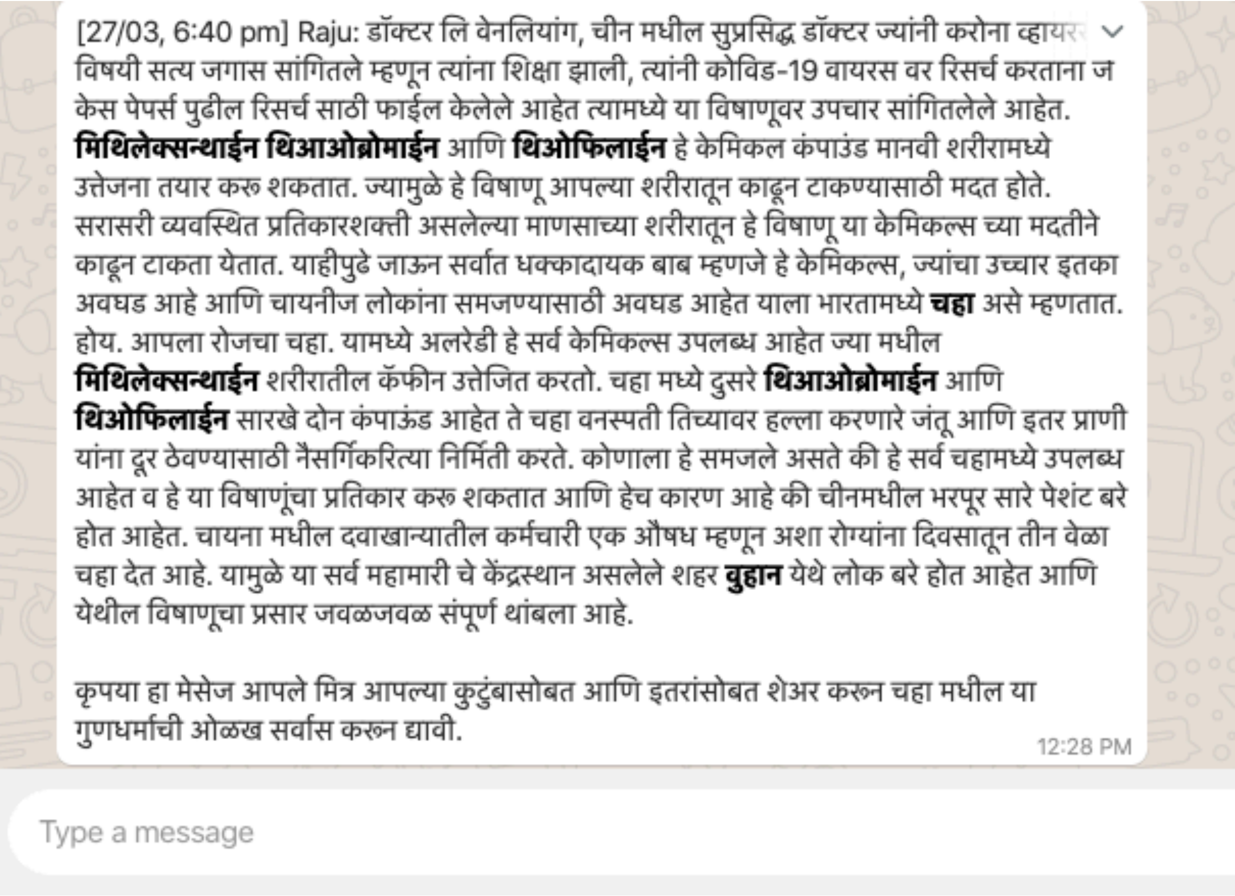

50. वरील मेसेज *

Mark only one oval.

- ( ) निश्चितपणे सत्य आहे
- ( ) कदाचित सत्य असेल
- ( ) कदाचित खोटे असेल
- ( ) निश्चितपणे खोटे आहे

Skip to question 51

व्हॉट्सअॅप वापर संबंधित माहिती

51. कोरोनाव्हायरस बद्दल आपण दररोज व्हॉट्सअॅप वरून किती मेसेज फॉरवर्ड करता? *

Mark only one oval.

- ० - २
- ३ - ५
- ६ - ८
- ८ पेक्षा जास्ती

52. कोरोनाव्हायरस बद्दल मेसेज व्हॉट्सअॅप वरून फॉरवर्ड करण्यापूर्वी त्यातील माहितीची खात्री करता का? करत असाल [illegible] किती वेळा? *

Mark only one oval.

- कधीच नाही
- १ - २५%
- २५ - ५०%
- ५० - ७५%
- ७५ - १००%

53. व्हॉट्सअॅप वरून मिळालेल्या माहिती आधारित कोरोनाव्हायरस विरुद्ध खालील प्रतिबंधात्मक उपयांविषयी आपलं कार मत आहे? *

Mark only one oval per row.

	ह्यावर विश्वास नाही	वापरून बघता येईल	१ - २ वेळा वापरलं आहे	नियमित वापर आहे व इतरांना सल्ला देत आहे
सोशल डिस्टंसिंग				
मास्क				
अॅलोपथीक (मेडिकल मधून घेतलेली) औषधे				
हर्बल औषधे				
आयुर्वेदिक औषधे				
होमिओपॅथीक औषधे				
घरगुती उपचार (गोमुत्र, लिंबाचे पाणी व सोडा, रसम)				

54. सध्याच्या कोरोनाव्हायरस साथीच्या माहितीबद्दल व्हॉट्सअॅप ह्या साधनाची उपयुक्तता आपल्या मते आहे की नाही? *

Mark only one oval.

- हो
- नाही

55. वरील उत्तराची काही कारणे?

This content is neither created nor endorsed by Google.

Google Forms
